# Emergence of a Novel Lineage and Wide Spread of a *bla*_CTX-M-15_/IncHI2/ST1 Plasmid among Nosocomial *Enterobacter* in Guadeloupe

**DOI:** 10.3390/antibiotics11101443

**Published:** 2022-10-20

**Authors:** Matthieu Pot, Yann Reynaud, David Couvin, Alexis Dereeper, Séverine Ferdinand, Sylvaine Bastian, Tania Foucan, Jean-David Pommier, Marc Valette, Antoine Talarmin, Stéphanie Guyomard-Rabenirina, Sébastien Breurec

**Affiliations:** 1 Transmission, Reservoir and Diversity of Pathogens Unit, Pasteur Institute of Guadeloupe, 97139 Les Abymes, France; 2 Laboratory of Clinical Microbiology, University Hospital Center of Guadeloupe, 97159 Pointe-à-Pitre, France; 3 Operational Hygiene Team, University Hospital Center of Guadeloupe, 97159 Pointe-à-Pitre, France; 4 Division of Intensive Care, University Hospital Center of Guadeloupe, 97159 Pointe-à-Pitre, France; 5 Faculty of Medicine Hyacinthe Bastaraud, University of the Antilles, 97157 Pointe-à-Pitre, France; 6 INSERM, Center for Clinical Investigation 1424, 97139 Les Abymes, France

**Keywords:** Caribbean, *Enterobacter cloacae* complex, ESBL, healthcare, *hsp60*, molecular sequencing, Nanopore, plasmid, ST114, ST1503

## Abstract

Between April 2018 and August 2019, a total of 135 strains of *Enterobacter cloacae* complex (ECC) were randomly collected at the University Hospital Center of Guadeloupe to investigate the structure and diversity of the local bacterial population. These nosocomial isolates were initially identified genetically by the *hsp60* typing method, which revealed the clinical relevance of *E. xiangfangensis* (*n* = 69). Overall, 57/94 of the third cephalosporin-resistant strains were characterized as extended-spectrum-β-lactamase (ESBL) producers, and their whole-genome was sequenced using Illumina technology to determine the clonal relatedness and diffusion of resistance genes. We found limited genetic diversity among sequence types (STs). ST114 (*n* = 13), ST1503 (*n* = 9), ST53 (*n* = 5) and ST113 (*n* = 4), which belong to three different *Enterobacter* species, were the most prevalent among the 57 ESBL producers. The *bla*_CTXM-15_ gene was the most prevalent ESBL determinant (56/57) and was in most cases associated with IncHI2/ST1 plasmid replicon carriage (36/57). To fully characterize this predominant *bla*_CTXM-15_/IncHI2/ST1 plasmid, four isolates from different lineages were also sequenced using Oxford Nanopore sequencing technology to generate long-reads. Hybrid sequence analyses confirmed the circulation of a well-conserved plasmid among ECC members. In addition, the novel ST1503 and its associated species (ECC taxon 4) were analyzed, in view of its high prevalence in nosocomial infections. These genetic observations confirmed the overall incidence of nosocomial ESBL *Enterobacteriaceae* infections acquired in this hospital during the study period, which was clearly higher in Guadeloupe (1.59/1000 hospitalization days) than in mainland France (0.52/1,000 hospitalization days). This project revealed issues and future challenges for the management and surveillance of nosocomial and multidrug-resistant *Enterobacter* in the Caribbean.

## 1. Introduction

*Enterobacter cloacae* complex (ECC) members are ubiquitous and are found as intestinal commensal bacteria [1]. The *Enterobacter* genus was reviewed and its taxonomy reclassified by genetic approaches such as identification of the 60-kDa heat shock protein partial coding gene (*hsp60*), and more recently, whole-genome sequencing (WGS). In 2018, ECC classification included 13 *hsp60* clusters (Hoffman’s approach) related to 22 phylogenetic clades (Sutton’s clades A–V) [1,2,3,4]. Phylogenetic analyses are ongoing; in 2020 and 2021, Feng Y. and colleagues illustrated the difficulty of accurate assignment based on a high computational approach, indicating possible important taxonomic updates. The authors identified 24 species and 22 novel *Enterobacter* genomospecies or taxons among this bacterial complex (see taxonomy-nomenclature in the Supplementary Data Set S1) [5,6].

These bacteria are included in the ESKAPE group as important opportunistic clinical pathogens that can harbor a wide range of resistance genes (*Enterococcus faecium*, *Staphylococcus aureus*, *Klebsiella pneumonia* complex, *Acinetobacter baumannii*, *Pseudomonas aeruginosa*, and *Enterobacter* spp.) [7]. The World Health Organization recently released a list of priority antibiotic-resistant organisms that included third-generation cephalosporin-resistant (3GC-R) ECC isolates [8]. Resistance in the *Enterobacter* genus is mediated mainly by the overexpression of the chromosomic *ampC* gene that codes for a cephalosporinase or is associated with the production of extended-spectrum-β-lactamase (ESBL) [1]. Plasmids are the main carriers of multiple resistance genes, including ESBL, in clinical strains and are an important vehicle for the horizontal transfer of resistance genes among *Enterobacteriaceae* [9]. Until now, it has been difficult to study these mobile genetic elements using high-throughput sequencing, as they assemble poorly in short-read sequencing (Illumina), and their genomic fragments (contigs) are difficult to distinguish from chromosomal fragments. Software tools with various analytical approaches have been developed to predict plasmid-related sequences in draft assemblies and plasmidomes in metagenomic data (coverage analysis, chromosomal and plasmid marker genes, *k-mer* frequencies signatures) [10,11,12,13]. Recent technological progress and developments in bioinformatics are also eliminating the barriers to plasmid genomics studies by combining, for example, high-throughput sequencing and Oxford Nanopore sequencing technology (ONT) to fully reconstruct plasmids [14].

In comparison with other *Enterobacteriacae*, such as the *K. pneumoniae* complex, few data are available on the prevalence of ECC in human infections in the French West Indies and other Caribbean islands [15,16,17]. In Guadeloupe, we previously studied the distribution of ECC members in local fruits and vegetables, wild fauna, livestock and human infections and found that ESBL-producing ECC were restricted to human isolates [18]. We also identified clinically relevant genetic lineages, such as ECC sequence type (ST) 114, in a continuum, from clinical settings (hospital effluents) to the natural receiving environment, treated wastewater and wild animals living near the treatment plant [19]. These elements suggest that the reservoir of ESBL-producing ECC is mainly human and that transfer from humans to animals and the environment can occur under significant anthropic pressure. Thus, within this continuum, a well-conserved plasmid (IncHI2/ST1) carrying the *bla*_CTX-M-15_ gene was found to be widespread among ESBL isolates assigned mainly to the ST114 lineage [19].

The primary objective of the present study was (i) to use WGS to identify the genetic background of ESBL-producing ECC collected from patients admitted to the University Hospital Center of Guadeloupe (UHCG) over 17 months. The secondary objectives were (ii) to study the genetic basis of 3GC-R ECC in order to investigate the spread of the well-conserved IncHI2/ST1 plasmid-carrying *bla*_CTXM-15_, and (iii) to determine the incidence of hospital-acquired ESBL-producing ECC associated with human infections and to compare it with that of major ESBL-producing *Enterobacteriacae*.

## 2. Results

### 2.1. Clinical Incidence of Hospital Acquired ESBL-Producing Enterobacteriaceae at the UHCG between 2018 and 2019

During the study period, a total of 330 ESBL-producing *Enterobacteriaceae* isolates associated with nosocomial infections was recorded at the UHCG. The most prevalent species among these epidemiological data were the *K. pneumoniae* complex (*n* = 218, 66.1%) followed by ECC (*n* = 72, 21.8%) and *E. coli* (*n* = 27, 8.2%). The other species were *Citrobacter freundii* (*n* = 5), *C. koseri* (*n* = 4), *K. aerogenes* (*n* = 3) and *Proteus mirabilis* (*n* = 1; details available in the Supplementary Data Set S2). Most of these ESBL-producing *Enterobacteriaceae* were isolated from urinary tract infection (*n* = 136, 41.2%), followed by catheter site infection (*n* = 68, 20.6%) and bacteremia (*n* = 62, 18.8%). The least represented infection sites were the respiratory tract (*n* = 24, 7.3%), surgical site (*n* = 19, 5.8%), and skin and soft tissue (*n* = 5, 1.5%). Sixteen strains were not associated with any of these infection sites (i.e., “Other” in Figure S1; details are provided in the Supplementary Data Set S2). Overall, the incidence of ESBL-producing *Enterobacteriaceae* was estimated at 1.59/1000 hospitalization days at the UHCG. This corresponded to incidences of 1.05 ESBL-producing *K. pneumoniae* complexes, 0.35 ECC and 0.13 *E. coli*. The samples were mainly isolated from men (*n* = 213; sex ratio = 1.84) and the median age of the patients was 65 years.

### 2.2. Enterobacter hsp60 Distribution and Antibiotic Resistance Patterns at the UHCG

During the same period, and irrespective of their resistance profile, a total of 135 ECC isolates associated with hospital-acquired infection were recovered and fully analyzed in this study. Associated patients were hospitalized in intensive care units (*n* = 48, 35.6%), surgical wards (*n* = 41, 30.4%), medical wards (*n* = 29, 21.5%), or emergency units (*n* = 15, 11.1%). Two strains were isolated from patients from unknown hospital departments. These bacteria were recovered from the urinary tract (*n* = 54, 40.0%), bacteremia (*n* = 23, 17.0%), catheter site infection (*n* = 19, 14.1%), surgical site infection (*n* = 15, 11.1%), respiratory tract (*n* = 7, 5.2%), or skin and soft tissue infection (*n* = 6, 4.4%). Eleven strains did not fit the different infection sites mentioned above (8.1%; “Other” in Figure S2, details are provided in the Supplementary Data Set S1).

With regard to the antibiotic-resistance phenotype of this bacterial collection, 41 hospital-acquired *Enterobacter* strains (30.4%) were characterized as wild-type against β-lactam antibiotics. The 3GC-R strains were associated with ESBL production (*n* = 57, 42.2%) or cephalosporinase overproduction without the ESBL gene (CoP, *n* = 37, 27.4%), of which one isolate (GENC133) was identified as a carbapenemase producer. Among these 94 3GC-R isolates, 44 (46.8%) were isolated from urinary tract infections. Various levels of resistance to fluoroquinolones, gentamicin and trimethoprim–sulfamethoxazole were observed in 3GC-R strains (Table S1). Most of the ESBL producers were resistant to these antibiotics (41/57, 72.0%). This specific bacterial collection reflected the clinical incidence of ESBL-producing ECC during the same period, as described in Section 2.1 (see details in Figure S2 and Supplementary Data Set S2).

**Figure 1:**
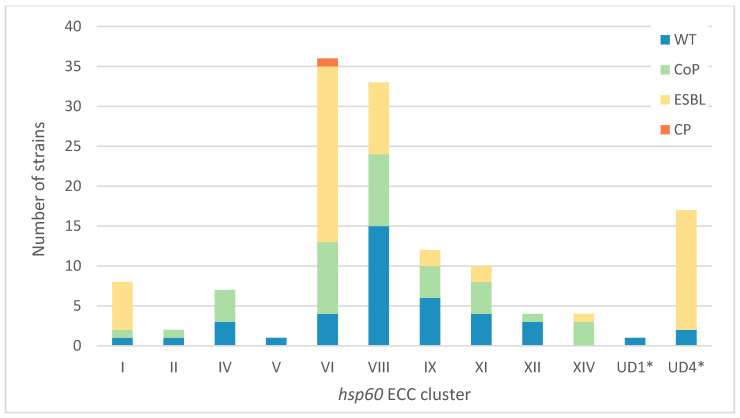
Distribution of 135 *E. cloacae* complex (ECC) members isolated from clinical samples according to the *hps60* approach. For each *hsp60* cluster recovered in this bacterial collection, nosocomial strains were grouped according to their resistance profile to β-lactam antibiotics: wild-type (WT), extended-spectrum-β-lactamase (ESBL), cephalosporinase overproduction without ESBL gene (CoP), or carbapenemase production (CP). The distributions in Figure S3 and the hsp60ECCtool output were identical (for details see Supplementary Data Set S1). Cluster XIV was defined according to Beyrouthy et al. and corresponds to *E quasihormaechei* [2,3,6]. * UD refers to “undefined *hsp60* cluster” [18]. They were not identified in the original paper of Hoffman et al. [4].

All these clinical isolates were clearly assigned to a Hoffman’s *hsp60* cluster. Assignation was first conducted with the approach proposed by the hsp60ECCtool and was confirmed according to the reference and bootstrap values in a phylogenetic tree (see Figure S3 and Supplementary Data Set S1). Hoffman’s clusters III, C-VII and C-XIII (sequence crowd) were not recovered among the 13 initial *hsp60* clusters of the *Enterobacter* genus. Overall, the *E. xiangfangensis*-related clusters were the most prevalent, with C-VI and C-VIII (*n* = 69, 51.1%; Figure 1 and Supplementary Data Set S1). Most of these isolates were characterized as 3GC-R (50/69, 72.5%). This observation was not cluster specific, however, and the proportion was not significantly higher than in other *hsp60* clusters (44/66, 66.7%, *p = 0.46*). In fact, undefined *hsp60* cluster 4 (UD4) accounted for 12.6% of the isolates (17 strains), and most of them were also 3GC-R (*n* = 15, 88.2%).

### 2.3. Specificities of ESBL-Producing ECC Populations

Because of the wide distribution of ESBL-producing strains among the ECC collection (57/135) and the similar antibiotic-resistance phenotype, these resistant strains were further analyzed (Table S1). WGS revealed that each *hsp60* cluster was correctly associated with a related Sutton’s clade. However, species prediction of the latest *Enterobacter* nomenclature, especially for UD4, is currently not accurate enough with the *hsp60* coding partial gene (see comparison on Supplementary Data Set S1). Pairwise average nucleotide identity (ANI) and in-silico DNA:DNA hybridization (isDDH) values against the different references are listed on Supplementary Data Set S1.

**Figure 2:**
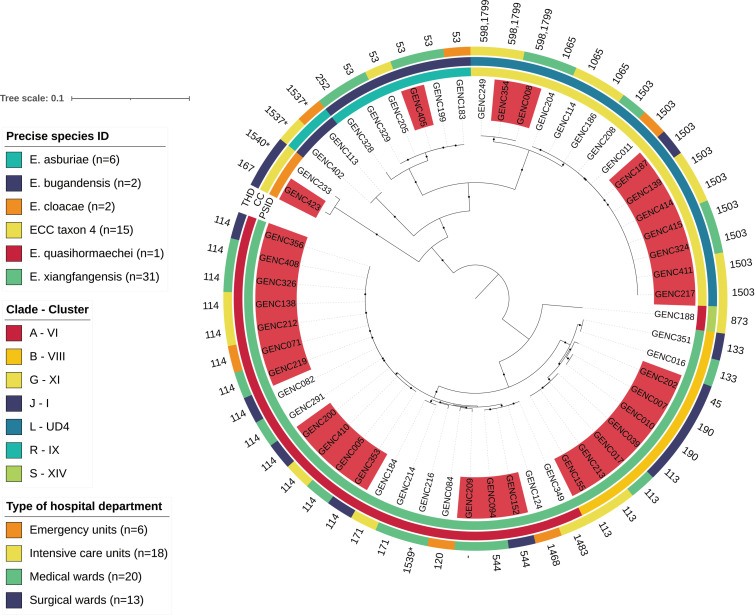
Maximum likelihood phylogenetic tree of clinical ESBL-producing *E. cloacae* complex (ECC) isolated between 2018 and 2019 in Guadeloupe. The core-genome phylogenetic tree was drawn from 57 genomes with iTOL. The tree was rooted at the mid-point and only bootstrap values equal to 100% are indicated by a black circle. The following metadata are indicated: the current species name (PSID) on the inner colored ring and the related ECC Sutton’s clade and *hsp60* cluster (CC) on the second one [3,4,6]. The type of hospital department (THD) is indicated on the last circle. Strains with a *bla*_CTX-M-15_ gene with a location predicted on a plasmid and an IncHI2/ST1 plasmid replicon are in red. * New sequence types (STs).

In-silico multilocus sequence typing (MLST) of the 57 whole-genome sequenced strains revealed limited diversity, as they belonged to 20 identified STs. Four novel STs were characterized in this ESBL-producing ECC collection (ST1468, ST1537, ST1539 and ST1540). Detailed profiles of the seven housekeeping genes used in the ECC MLST are presented in Supplementary Data Set S1. The most prevalent were ST114 (*n* = 13, 22.8%) and ST1503 (*n* = 9, 15.8%), associated with *E. xiangfangensis* and ECC taxon 4, respectively. They were followed by ST53 (*n* = 5, *E. asburiae*) and ST113 (*n* = 4, *E. xiangfangensis*; Figure 2 and Supplementary Data Set S1). These four major STs were isolated mainly from urinary infections (18/31, 58.1%). They were mainly identified in medical wards and intensive care units (*n* = 22, 71.0%).

Precise core-SNP analyses among this selection of major STs revealed a low number of SNPs between some strains as suggested for ST114 (mean SNPs difference between isolates, *n* = 46.3), ST1503 (*n* = 16.9), ST53 (*n* = 38.8) and ST113 (*n* = 13.3). More specifically, SNPs’ different count matrix indicated the presence of bacterial clones especially for ECC taxon 4 ST1503 (clonality was defined as ≤ 10 pairwise SNPs in the core-genome; Supplementary Data Set S1). As these STs were recovered from different wards at the UHCG (Figure 2), the SNP values suggest that an environmental reservoir was responsible for the direct or long-term cross-transmission of ESBL-producers during this 17-month study.

### 2.4. Sequence Analyses and Synteny of Major bla_CTXM-15_/IncHI2/ST1 Plasmids

Most of the ESBL-producing ECC isolates harbored a *bla*_CTX-M-15_ gene (98.2%, 56/57), and the last one contained a *bla*_GES-7_ determinant (GENC084; Supplementary Data Set S1). With prediction tools, we classified most of the *bla*_CTX-M-15_-carrying contigs, with 80.4% (*n* = 45) predicted at the same location with three types of software. The others were predicted at the same location with an acceptable percentage identity with two types of software (*n* = 7), and only four isolates were not retained for this *bla*_CTX-M-15_-carrying analysis (different outcomes, percentage identity < 70.0% when applicable). In this ESBL ECC collection, the CTX-M-15 coding gene was predicted mainly on plasmid sequences (39/52, 75.0%). Its chromosomal location was predicted for various *Enterobacter* species, including *E. xiangfangensis* (8/13), *E. asburiae* (*n* = 2), ECC taxon 4 (*n* = 2) and the two *E. bugandensis* ST1537 (see Supplementary Data Set S3).

In addition to the prediction data, some genetically unrelated strains presented similar antibiotic resistance gene patterns, as previously observed in local ST114 lineage [19]. In the present study, in addition to genes coding for chromosomal AmpC, the *bla*_CTX-M-15_ determinant and the efflux pumps [m*df(A)*, *oqxA/oqxB*], genes that confer resistance to aminoglycosides [*aac(3)-IIa, aac(6′)-Ib-cr, aph(3″)-Ib, aph(6)-Id*], β-lactam class [*bla*_OXA-1_, *bla*_TEM-1B_], fluoroquinolones [*aac(6′)-Ib-cr, qnrB1*], fosfomycin [*fosA*], sulfonamides [*sul2*], tetracyclines [*tet(A)*] and trimethoprim [*dfrA14*] were present in more than 60.0% of the strains. A truncated *catB3* gene was also found (Table S2 and Supplementary Data Set S1). These genes were identified in all the recovered species except for *E. bugandensis* and *E. quasihormaechei*, which suggests carriage by a similar plasmid(s). Most of these strains presented PlasmidFinder and pMLST outputs, indicating the presence of an IncHI2/ST1 plasmid replicon (*n* = 36); the allele profiles did not permit the determination of IncHI2 ST for five strains (see Supplementary Data Set S3). A Bowtie reconstruction and mapping with previously sequenced plasmid pGENC200 (associated strain: GEN200; CP061495; 291,493 bp) as the reference also suggested the possible carriage of these genes on a similar mobile genetic element, with an 82.7–96.6% similarity in identity among the other ESBL-producing isolates with an IncHI2 signature (Table S3). Finally, among other resistance genes, we reported that two *E. xiangfangensis* strains (ST171; GENC184, GENC214) possessed the *mcr-9* gene but lacked the associated regulatory genes *qseC/qseB* [20].

**Figure 3:**
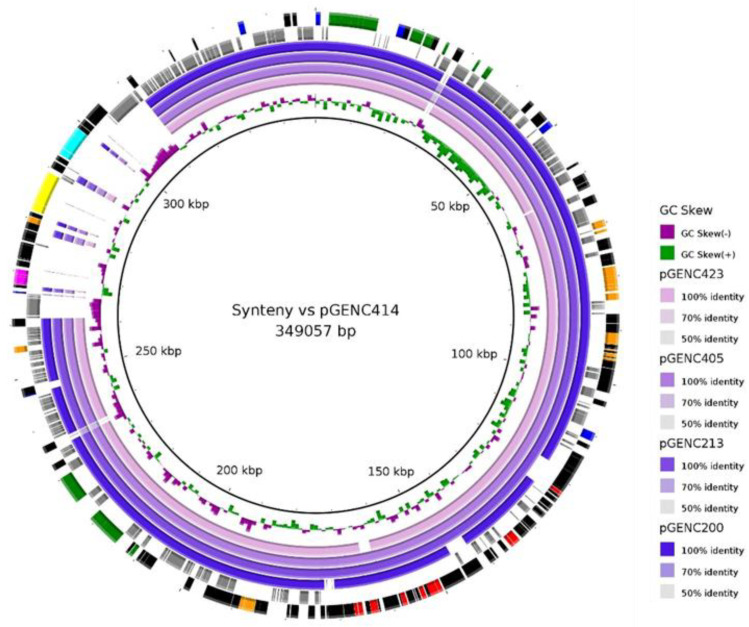
Syntenic analysis of five *bla*_CTX-M-15_/IncHI2/ST1 plasmids from the UHCG. This figure was developed with BRIG software. The first black ring represents the pGENC414 plasmid, used as the reference, and the second ring indicates the GC skew. This first plasmid was isolated from the *E. cloacae* complex taxon 4 ST1503 (GenBank accession number OL331020, recovered in July 2019). The inner to the outer rings correspond to the following pairwise comparison with IncHI2/ST1 plasmids from other ECC STs isolated at the same hospital: pGENC423 (*E. cloacae*, ST167, OL331021, August 2019), pGENC405 (*E. asburiae*, ST53, OL331019, May 2019), pGENC213 (*E. xiangfangensis*, ST113, OL331018, November 2018) and pGENC200 (*E. xiangfangensis*, ST114, CP061495, October 2018). The last rings represent the genetic map of pGENC414, with the first hypothetical proteins shown as grey arrows, followed by coding sequences in black arrows, except for genes associated with antibiotic resistance (red), conjugation (green), formaldehyde detoxification (fuchsia), heavy-metal resistance (orange), Fe(III) transporter system (yellow), *lac* operon (aqua) and plasmid replication, partition or maintenance (blue). The genetic details and metadata of the plasmids are shown in Supplementary Data Set S3.

After the reads mapping analysis with previously sequenced pGENC200, a more precise comparison was performed with additional Illumina and Oxford Nanopore hybrid assemblies. We compared four novel local circularized plasmids recovered from different *Enterobacter* species to investigate the possibility of an “epidemic plasmid” in our clinical isolates. BLAST Ring Image Generator (BRIG) and MAUVE software tools revealed strong similarity in their backbone structure (Figure 3 and Figure S4). Calculation of the Mash distance for plasmid similarities led to a similarity mean of 5.6 × 10^–3^ (see Supplementary Data Set S3). The insertion of various additional regions into these plasmids resulted in variable lengths (from 291,493 to 349,057 bp), including an Fe(III) transporter system in the larger plasmid pGENC414 (Figure 3 and Supplementary Data Set S3). These plasmids encoded not only resistance genes to several families of antibiotics but also the determinants of resistance to heavy-metal ions including arsenic [*ars*], mercury [*mer*, *tni*] and tellurium [*ter operon*], and also a toxin–antitoxin system (Figure 3 and Supplementary Data Set S3). Finally, the plasmid taxonomic unit (PTU) of these local circularized plasmids was -HI2, which corresponds to the conjugative plasmid with a bacterial host range value of IV according to Redondo-Salvo et al. [21,22].

### 2.5. Literature Search for Similar IncHI2/ST1 Resistance Plasmids

In parallel to this local analysis, a total of 91 similar IncHI2/ST1 plasmids with complete metadata were recovered from the Plasmid database (PLSDB; Mash distance min: 0.0021891; max: 0.0287714 against the largest pGENC414) [23]. Details of the output are provided in Supplementary Data Set S3. Among them, 98.9% presented -HI2 PTU (90/91). These large mobile genetic elements were associated with the genus *Enterobacter* (*n* = 49, 53.8%), followed by *Salmonella* (*n* = 12, 13.2%) and *Citrobacter* (*n* = 11, 12.1%). The other genera, including *Cronobacter*, *Escherichia*, *Klebsiella, Leclercia*, *Phytobacter* and *Raoultella*, were detected less than ten times each. These *Enterobacteriaceae* were mainly associated with human (*n* = 57, 62.6%) and domestic animal (*n* = 15, 16.5%) samples. The remaining 19 IncHI2/ST1 plasmids were isolated from the environment (*n* = 6), food products (*n* = 5), wild animals (*n* = 5) and various surface samples (*n* = 5). They were recovered and sequenced from all over the world; specifically, East Asia (*n* = 25, 27.5%), Europe (*n* = 25, 27.5%) and North America (*n* = 18, 19.8%). The antibiotic resistance genes carried by these IncHI2/ST1 plasmids were varied and only seven showed the same pattern as observed in our clinical setting, including pGENC200 (CP061495) and three plasmids previously recovered from animals living near the wastewater continuum of the UHCG (CP061496, CP061494, CP061493). The three other highly similar plasmids were recovered in Kenya (CP021463) and South Korea (CP024813) in human samples, and in Switzerland (CP048350) in the environment. These IncHI2/ST1 plasmids were also frequently associated with ESBL production, such as *bla*_SHV-12_, (*n* = 28, 30.8%) and *bla*_CXT-M_ determinants (*n* = 15, 16.5%). In addition, gene families encoding carbapenemases were identified, including *bla*_VIM_ (*n* = 16, 17.6%), followed by *bla*_IMP_ (*n* = 4), *bla*_KPC_ (*n* = 3) and *bla*_NDM_ (*n* = 3). Among other resistances, we noted the presence of the complete *ter* operon in 90 plasmids (98.9%). The *mcr-9* gene was identified in 62 sequences (68.1%) but only 13 were associated with the *qseC/qseB* regulatory genes [20].

### 2.6. Phylogenetic Analysis of ECC Sutton’s Clade L in Guadeloupe and Global Comparison

Due to the uncommon prevalence of ESBL-producing Sutton’s clade L in our collection (i.e., UD4 *hsp60* cluster in Figure 1 and Figure 2), we compared it with international and other local WGS associated with this vernacular name [3,24]. Overall, the ECC Sutton’s clade L WGS contained 58 isolates with complete metadata. Local ECC Sutton’s clade L isolates comprised 16 from the present clinical study, 3 from screening in the UHCG and the urban wastewater continuum and 1 obtained in the natural environment [18,19,25]. Most of the 38 international strains retained for this analysis were isolated from humans (*n* = 36, 94.7%), except for one collected from a pig (PCFC00000000) and one isolated in activated sludge (BMBZ00000000). Overall, these strains were recovered in 12 geographic areas, with the oldest having been isolated in 2006 in the USA (LEDT00000000; Supplementary Data Set S4). The antibiotic resistance profiles indicated a high prevalence of 3GC-R strains in the non-Guadeloupean collection (21/38, 55.2%), with *bla*_CTX-M_ genes identified in ten strains, of which eight were *bla*_CTX-M-15_. However, the local InchI2/ST1 replicon and associated resistance pattern were not identified in other ECC Sutton’s clade L isolates (Supplementary Data Set S4).

**Figure 4:**
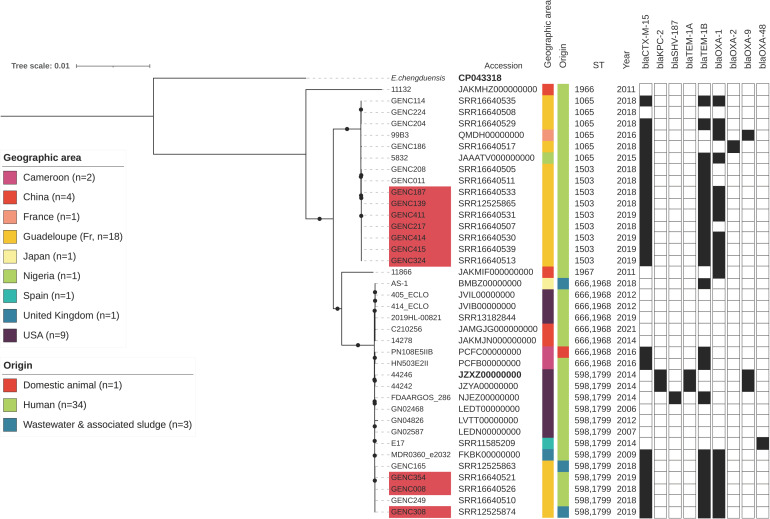
Maximum likelihood phylogenetic tree of *E. cloacae* complex (ECC) taxon 4 isolated in Guadeloupe (*n* = 18) and internationally (*n* = 20). The core-genome phylogenetic tree was drawn with iTOL, from 38 genomes recovered worldwide between 2006 and 2021 [24]. The tree was rooted with *E. chengduensis* reference (in bold: CP043318) [5,26]. ECC taxon 4 reference is also presented in bold (JZXZ00000000) [5], and only bootstrap values equal to 100% are indicated by a black circle. The following metadata are indicated: date of isolation, sequence type (ST) and antibiotic resistance genes in black squares. Only acquired genes that confer resistance to β-lactam antibiotics were included. Strains with a *bla*_CTX-M-15_ gene with a location predicted on a plasmid and an IncHI2/ST1 plasmid replicon are shown in red. Metadata are shown in Supplementary Data Set S4.

In the latest taxonomic analysis, ECC Sutton’s clade L was divided into two *Enterobacter* members (i.e., *E. chengduensis* and ECC taxon 4; see taxonomy-nomenclature in the Supplementary Data Set S1) [3,5,6]. Phylogenetic analysis of this clade revealed clear clustering into three main branches (Figure S5). The first contained seven international strains belonging to ST414 and two strains from Guadeloupe with novel ST1533 and ST1535 (additional representation in our reference [25]). All these isolates were recovered from humans except JAKLRZ000000000, and were referred as to as *E. chengduensis* [5,26]. The other two major branches corresponded to ECC taxon 4 (*n* = 38; see Figure S5 and Supplementary Data Set S4) [5]. Most of ECC taxon 4 isolates belonged to ST598,1799 (13/38, 34.2%; Figure 4). In the Guadeloupean samples, this ST contained two ESBL-producing isolates recovered in wastewater from the UHCG in 2018 and 2019 (SRR12525863 and SRR12525874) and three ESBL-producing strains isolated from patients during the same period [19]. Most of the clinical ECC taxon 4 from Guadeloupe, however, belonged to the recently defined ST1503 (9/18, 50.0%) and formed a cluster with ST1065, which at present is only associated with human infections (Figure 4). Comparison with international ECC taxon 4 indicated that the predominant ST identified at the UHCG is currently limited throughout the island (Figure 4 and Supplementary Data Set S4).

## 3. Discussion

### 3.1. Nosocomial Incidence of ESBL-Producers in Guadeloupe

This 17-month epidemiological study at the UHCG showed a higher incidence of ESBL-producing *Enterobacteriaceae* (1.59/1000 hospitalization days) than in mainland France in 2018 (0.52) [27]. Among ESBL-producing *Enterobacteriaceae*, the incidences of *K. pneumoniae* complex (1.05) and ECC (0.35) were higher and that of *E. coli* (0.13) was lower than in mainland France. The predominance of *E. coli* among ESBL-producing *Enterobacteriaceae* was identified in a national inventory in 2012 [28]. These findings indicate probable epidemiological specificities of the island [27]. The French National Spares surveillance system indicated that the incidence of ESBL-producing ECC in 2018 was 0.05, lower than that of *K. pneumoniae* complex (0.17) and *E. coli* (0.27), and similar observations were reported from other national surveillance systems [29].

### 3.2. The Local hsp60 Cluster Distribution of Clinical ECC Does Not Mirror That in the National Population

There is no clear phylogenetic consensus in the recently published research papers associated with this bacterial complex (example references [30,31]). Because of the extensive changes in ECC assignment due to the evolution of bioinformatics and genetic methods, we used various taxonomic approaches and vernacular names to link previous, present, and future works on this genus [3,4,6]. We first investigated the *hsp60* cluster distribution of clinical ECC isolates from inpatients at the UHCG [4]. It was concordant with several European studies, as highlighted by the high proportion of successful C-VI and C-VIII. *E. xiangfangensis*, which is the related opportunistic species, was isolated at various sites of infection, including invasive samples, with the urinary tract being the most common [32,33,34]. This high proportion of C-VI and C-VIII was also recently identified in East Asia [31,35]. In contrast, we noted several local specificities. Cluster III (*E. hoffmannii*) was not represented in this French West Indies island, while it has been frequently reported in hospitals in metropolitan France [32,36]. Furthermore, the third most represented *hsp60* cluster was named UD4 in our previous local analysis and is associated with Sutton’s clade L [3,18]. In addition, other recent surveys suggested geographical variations in ECC distribution in human infections: for example, C-II (*E. kobei*) was one of the most represented in China [35], and C-IV (*E. roggenkampii*) in Spain [37]. Larger analyses involving different countries and geographical areas with an identical protocol are needed to clearly assess these variations.

To date, misidentifications of UHCG have had no impact on antibiotic therapy, as the clinical strains identified as belonging to the ECC had similar antibiotic resistance patterns. The emergence and rapid spread of resistant plasmids and specific lineages in the future could, however, have a direct impact on antibiotic therapy and cause large nosocomial outbreaks globally. As an accurate ECC identification is crucial for monitoring outbreaks, our observations underline the usefulness of the *hsp60* typing approach, an inexpensive initial screening tool. Although this method as defined by Hoffman and Roggenkamp is limited (i.e., single gene fragment analysis), it could be updated, as it provides a more accurate characterization of *Enterobacter* diversity than traditional approaches and seems to be a good first step for preliminary comparison in hospital settings [4,18,32,33,35,36,37]. The hsp60ECCtool developed here provides a more evident identification of *hsp60* clusters in the ECC than a direct comparison using BLASTn against the entire National Center for Biotechnology Information (NCBI) database. Like other recently proposed methods and markers, this updated approach should help the scientific community to rapidly sort and classify ECC members for epidemiological and screening purposes [38,39].

### 3.3. Dissemination of Resistant Lineages through Hospital Wards

The ESBL producer sequencing indicated the circulation of several specific lineages at the UHCG. Some members of the ECC have been recognized as nosocomial pathogens, and the worldwide diffusion of specific lineages harboring a large panel of antibiotic resistance genes is being characterized. Clinical and veterinary analyses showed that ST113, ST114, ST133 and ST171 belonging to *E. xiangfangensis*, as identified in our study, are successful resistant clones [34,40,41,42,43,44]. In addition, ECC ST114, which predominated in this collection, is often associated with *bla*_CTX-M-15_ in the literature [19,40,43,44]. This ESBL determinant was also the most frequent in Guadeloupe [15,18,19]. A large, curated Data Set initially provided by Wu et al. in 2020 indicated that the associated *Enterobacter* species was the most sequenced since 1987 (831/1544; 53.8%). *E. xiangfangensis* has been recovered from various compartments, including human samples (759/1354; 56.1%; see Supplementary Data Set S5) [5,24]. As the authors point out, the reason for its high prevalence worldwide is not clear and further studies are needed [5].

The second most prevalent ESBL-producing lineage, ST1503, appeared to be well conserved throughout the sampling period and associated strains were isolated from patients in various wards. This ST was initially associated with the Sutton’s clade L. By performing a comparison with internationally published genome collections, we identified a few isolates within this Sutton’s clade that are also referred to as *E. chengduensis* and ECC taxon 4 according to the most recent nomenclature (see taxonomy-nomenclature in the Supplementary Data Set S1) [3,5,24,26]. Human infections caused by these two ECC members have rarely been reported. They represented only 0.9% of the NCBI curated Data Set in 2019 (14/1544; Supplementary Data Set S5) [5,24]. To our knowledge, this is the first description of the nosocomial incidence of ECC taxon 4 (17/135, 12.6%), and ST1503 was not found outside the island. This finding underlines the importance of a complete characterization of strains related to ECC taxon 4 in the future. A first name for this taxon was indicated in 2020 on NCBI; however, additional research and validation have not been carried until now (SAMD00239574: *Enterobacter kurensis*) [24].

Among the other clinically relevant STs in our collection, ST53 (associated with *E. asburiae*) was initially described from clinical strains in Japan and is of growing importance due to carbapenemase production [45], as also mentioned for ST873 (*E. quasihormaechei*) [2,5]. In the global, curated Data Set from NCBI, *E. asburiae* represented 4.7% of the genome collection (73/1544), and only nine strains have been identified to be *E. quasihormaechei* since 2014 (Supplementary Data Set S5) [5,24]. No additional carbapenemase-producing *Enterobacter* were identified at the UHCG during the study period. As the only carbapenemase-producing strain found was ST114 [18], its presence should be monitored to avoid its spread. In other countries, the presence of carbapenemase-producing ECC and related STs is well documented and remains a growing public health issue [37,40,42,45,46,47]. Among resistance genes against “last resort” antibiotics, the *mcr-9* signature identified in two ECC ST171 strains is also of concern due to its emergence in different *Enterobacter* lineages and plasmidic diffusion in other compartments [48,49,50]. In-silico analysis did not, however, identify the *qseC/qseB* system, which is generally associated with the up-regulation of this gene [20].

The isolation of various resistant lineages during the study period indicates possible long-term transmission, with sporadic presence and persistence in various wards. Further studies should be conducted to understand the ECC transmission pathways at this hospital (patient to patient, environment to patient), and specific infection control measures should be proposed to reduce in-hospital transmission [2,30,45,46]. This would not, however, reflect the nosocomial situation in other medical centers on the island. Because of extensive communication among the Caribbean islands, data collection must be expanded to determine whether specific lineages such as ST114 and ST1503 circulate and persist as hospital-associated ESBL-producing strains, as described recently for the *K. pneumoniae* complex in some Caribbean states [16]. Previous work in Ireland and the United Kingdom indicated the wide dissemination of some ECC clones and inter-hospital connectivity [34]. A similar observation was made for a successful ESBL-producing clone in China (ST591) [35]. Until now, the human compartment remains the most represented in *Enterobacter* whole-genome data (Supplementary Data Set S5) [5,24]. The use of a wider “one health” approach is important for investigations regarding possible circulation and persistence in the environment and local community carriage.

### 3.4. A Successful bla_CTXM-15_/IncHI2/ST1 Plasmid Is Circulating in ECC Nosocomial Populations

With a mapping approach, followed by long-reads and hybrid assembly, we confirmed plasmid-borne dissemination of an antibiotic resistance gene pattern associated with the local development of several ECC members. The high prevalence of a well-conserved *bla*_CTX-M-15_/IncHI2/ST1 plasmid in various *Enterobacter* suggests a key role in the dissemination of novel lineages (example, ST1503). An IncHI2 signature has frequently been identified in ECC members in clinical and veterinary studies worldwide and was recently associated with carbapenemase determinants [2,28,34,35,44,51]. Its important association with the *Enterobacter* genus, also observed with the PLSDB data [23], remains unclear and should be explored further (see Supplementary Data Set S3).

This plasmid type is well known to be present in a wide bacterial range and could play an important role in the maintenance of resistance [9,52]. In addition to data from PLSDB presented here [23], the taxonomic classifier of plasmids tool (COPLA) output highlighted that similar local and international plasmids had a host range value of IV (-HI2 PTU) [21]. This value indicates that mobile genetic elements can spread among *Enterobacteriaceae* and also species of the *Erwiniaceae*, *Morganellaceae* and *Yersiniaceae* families [22]. Fully sequenced IncHI2/ST1 plasmids obtained from ESBL-producing ECC from local wastewater and various animal taxa showed a high percentage similarity with international plasmids and raised concern about their transfer though various compartments, hosts and bacterial species [19]. Conjugative transfer through other *Enterobacteriaceae* species has been clearly demonstrated (pEB-247, LN830952) [51]; however, this event was thermosensitive for this incompatibility group, with a low range of temperature efficacy globally < 30 °C [9,51]. The regulation of conjugation of this incompatibility group is being elucidated. In the latest studies of Gibert et al., the expression of a membrane protein (TrhR) and a cytoplasmic protein (TrhY) encoded in the plasmid was associated with the activation of transfer operons (associated with *tra* genes) and by a cascade effect, with initiation of its conjugation. The action of these two proteins is, however, regulated by different mechanisms. The HtdA protein partially counteracts the activity of TrhR/TrhY at low temperatures, whereas, at higher temperatures (37 °C), overexpression of an H-NS/Hha protein complex represses the gene encoding the TrhR protein and blocks initiation of transfer [53,54]. This specificity is in accordance with environmental conjugation but not in the mammalian gut.

This plasmid presents a toxin–antitoxin system and high genetic plasticity. For instance, the Fe(III) transporter system was also identified in a large, similar IncHI2/ST1 (pKST313-UGA14, CP021463; 352,906 bp) plasmid recovered from a *Salmonella enterica* strain isolated in Kenya in 2003 [52]. Like this previously sequenced mobile genetic element, pGENC414 also contains a complete *lac* operon and formaldehyde detoxification system but not the region with genes related to sucrose catabolism [52]. This large “epidemic” plasmid type has numerous genes that encode formaldehyde detoxification and resistance to various antibiotic families and heavy metal ions, so that its hosts can develop in unfavorable conditions, such as wastewater and contaminated streams, due to the large exposome [19,55,56]. Recent work on *Klebsiella* and tellurite resistance shows that this operon is clearly associated with infection and could enhance fitness in the gut during colonization [57]. Further research is necessary to clarify this successful link with the *Enterobacter* genus. Bacterial persistence in the hospital environment without specific selective pressure is, however, still undefined. Well-adapted bacteria in this environment could be major contributors to nosocomial outbreaks and the maintenance of this resistant plasmid and vice versa [2,46,58]. In our study, the entire *ter* operon (*terZABCDE*) was identified on 97.6% of ESBL producers with an IncHI2 replicon (40/41) and on only two strains without this replicon (2/17; 11.8%) [59]. In the similar IncHI2/ST1 plasmids recovered from PLSDB, 98.9% were also *ter* operon positive. The screening of the nosocomial environment with selective agar, supplemented with tellurite and antibiotics, could help to identify *Enterobacter* reservoirs with the *bla*_CTX-M-15_/IncHI2/ST1 plasmid. Similar approaches have been developed for shiga toxin-producing *E. coli*, also known to be associated with the *ter* operon on its chromosome [60].

### 3.5. Limitations of the Study

The COVID-19 pandemic may have influenced the epidemiological dynamics of antibiotic resistance genes and associated plasmids or bacterial lineages. Our pre-COVID-19 study would be a useful resource for further analysis to assess this potential impact in hospitals and especially the associated *Enterobacter* population structure. However, this project has several limitations. It has a relatively small sample size over a 17-month period. Additional analyses were provided on the incidence of nosocomial ESBL producers to assess the representativeness of the bacterial collection and to overcome this problem. Although convenient, the initial partial sequence analysis of *hsp60* does not allow for the thorough delineation of some species, as observed here for *E. chengduensis* and ECC taxon 4. Full sequencing of this region could, however, refine this identification and further analyses are required (Supplementary Data Set S1 and S4). The spread of the *bla*_CTX-M-15_ and other resistance genes has been observed at the UHCG and cannot be generalized to other institutions on the island. In addition, no whole-genome sequencing has been performed on other ESBL *Enterobacteriacae* such as the *K. pneumoniae* complex and on the hospital environment in order to assess the dissemination of lineages and efficient plasmids.

## 4. Materials and Methods

### 4.1. Collection of Metadata on ESBL Enterobacteriaceae Associated Infections

In order to compare ECC with other relevant bacteria, an initial investigation of the incidence of nosocomial ESBL *Enterobacteriaceae* acquired at the UHCG was performed between April 2018 and August 2019. In this study, we followed the definition of nosocomial infections provided by the World Health Organization. They correspond “to infections acquired during hospital care which are not present or incubating at admission. Infections occurring more than 48 h after admission are usually considered nosocomial” [61]. All clinical metadata on ESBL *Enterobacteriaceae* that meet this definition were collected during this 17-month period. Community-acquired ESBL *Enterobacteriaceae* infections were thus excluded. Isolates from systematic colonization screening were not analyzed; furthermore, if more than one isolate belonging to the same species with the same antibiotic susceptibility pattern was recovered from the same patient, only the first was included. For each strain, the date of admission, the hospital ward, the date of a positive specimen and the nosocomial infection site were recorded. The incidence of ESBL *Enterobacteriaceae* infection was calculated per 1000 in-hospital patient days (Supplementary Data Set S2).

### 4.2. Collection of Clinical ECC Strains and Associated Data

In parallel, non-duplicate ECC isolates were collected at the laboratory between April 2018 and August 2019 from patients admitted to the UHCG. Only infectious isolates considered to be hospital-acquired according to the same definition given in Section 4.1 were conserved (*n* = 135). The study protocol was approved by the local ethics committee (reference A5_19_12_05_TRAMID). Briefly, after isolation, bacteria were found to be related to the “*E. cloacae* complex” by performing matrix-assisted laser desorption/ionization time-of-flight mass spectrometry and associated software (MALDI-TOF MS; bioMérieux, Marcy L’Etoile, France) [62]. Antimicrobial susceptibility was then analyzed as recommended in the 2018 CA-SFM/EUCAST guideline by the disc diffusion method using the Mueller–Hinton medium [63]. As most of the C3G-R *Enterobacter* overproduced their cephalosporinase, an additional investigation was conducted with Mueller–Hinton medium supplemented with cloxacillin to detect ESBL production [63]. The resistance profile of these ECC isolates was characterized against β-lactam antibiotics as follow: wild-type cephalosporinase (WT), cephalosporinase over-production (without ESBL production; CoP), ESBL or carbapenemase producer. The sampling date, the nature of the biological sample and the hospital ward were recorded anonymously. Antimicrobial susceptibility was determined for the following antibiotic components: ampicillin (10 μg), amoxicillin–clavulanic acid (20–10 μg), ticarcillin (75 μg), temocillin (30 μg), cefoxitin (30 μg), cefotaxime (5 μg), ceftazidime (10 μg), aztreonam (30 μg), cefepim (30 μg), ertapenem (10 μg), gentamicin (10 μg), amikacin (30 μg), nalidixic acid (30 μg), ciprofloxacin (5 μg), tigecycline (15 μg) and trimethoprim–sulfamethoxazole (1.25–23.75 μg; Supplementary Data Set S1).

### 4.3. DNA Extraction and hsp60 Cluster Identification

In addition to routine species identification of ECC members by MALDI-TOF MS, partial *hsp60* coding gene amplification and sequencing were conducted for new isolates, as previously described [4,18]. All amplicons were first entered into the hsp60ECCtool v1.1.0, available on our Galaxy server [64,65,66]. It allows for the easy identification of ECC at the *hsp60* cluster level and a species prediction with the BLASTn approach and specific databases. The tool includes a large panel of complete, non-redundant sequences that code for the *hsp60* extracted from ECC assemblies recovered in GenBank (July 2021, 194 sequences) [67]. These references were sorted and identified at species, Sutton’s clade and Hoffman’s cluster levels [2,3,4,5,18]. In addition, a maximum likelihood phylogenetic reconstruction was performed with IQ-tree v2.1.2 and associated dependencies (–merit BIC: TIM3e+I+G4 –ufboot 1000 –bnni) [68,69,70], after alignment with MAFFT and a large reference panel (-flavor auto, v7.505; see Figure S3 legend for details) [18,71]. The phylogenetic tree was then drawn and annotated with the online tool iTOL v6.4.2 (Figure S3) [72]. This extended reference panel was used to avoid any “atypical clustering”, as previously mentioned [32]. DNA was initially extracted with the QIAamp DNA minikit (Qiagen, Hilden, Germany).

### 4.4. Assembly and Core-Genome Phylogenetic Analyses of ESBL ECC

To investigate the genomic dynamics of ECC members and their mobile genetic elements, WGS was conducted on all the recovered strains characterized as ESBL producers, except for the 23 previously sequenced isolates (for details of the new collection see Supplementary Data Set S1) [18,19]. These strains were sequenced at the “Plateforme de microbiologie mutualisée” on the Pasteur International Bioresources network (Institut Pasteur, Paris, France). The method and software used in the short-read sequencing step were the same as those previously described [18,19]. We obtained an estimated coverage of 93-fold (AlienTrimmer v0.4.0) [73]. SPAdes software v3.12.0 was used for de novo assembly (-careful), and quality was verified with QUAST v5.0.2 [74,75]. This step led to a N50 mean of 172,235 (min: 79,193; max: 323,676). The mean benchmarking universal single-copy orthologs (BUSCO) score was estimated as 98.7% of completeness (single-copy; lineage_Data Set_Enterobacterales; v5.0.0) [76].

The rest of our bioinformatics methodology is presented in a supplementary schematic diagram (Figure S6). In order to follow the chronology of taxonomic changes in the genus *Enterobacter*, phylogenetic identification was first confirmed by performing ANI with FastANI software v1.3 against the ECC clade references proposed by Sutton et al., in 2018 (*n* = 22), with cut-off values of >95.0% for species delineation [3,77]. In parallel, we conducted a precise species identification according to Wu et al. [5]. This second approach proposed in 2020 includes isDDH and ANI analyses against a most-important reference panel (ANI ≥ 96.0% cut-off; *n* = 46). The isDDH value estimated with the online genome-to-genome distance calculator tool (GGDC v2.1) was a ≥70.0% cut-off for species delineation (pairwise analyses against all *Enterobacter* references are provided in Supplementary Data Set S1) [5,6,78].

To observe the genomic structure of the collection of ESBL-producers, assembled and identified genomes were annotated with Prokka software v1.14.6 [79], and a global core-genome alignment was carried out with Roary v3.13.0 (-cd FLOAT 95) [80]. These steps led to a global alignment of 2,133,125 bp, with 2098 genes shared by a least 95.0% of bacterial isolates. ML phylogenetic reconstruction was performed with IQ-tree and associated dependencies (–merit BIC: GTR+F+R8 –ufboot 1000 –bnni) [68,69,70]. The tree was drawn and annotated with iTOL (Figure 2) [72].

### 4.5. Sequence Type and Precise Clonal Relatedness among Major Enterobacter

For all WGS analyses, MLST was performed with mlst software v2.19.0 against PubMLST [81,82,83]. As this bacterial complex includes different species, a deeper analysis to estimate the number of SNPs between members of the major ESBL-producing STs recovered during this study was performed. To conduct further analyses, four strains were selected from different ECC members and fully sequenced by performing ONT long-read sequencing (GENC213, GENC405, GENC414 and GENC423). The long-reads sequencing methodology was the same as described previously [19]. Unicycler software v0.4.8 was used to generate hybrid assemblies between Illumina and the Nanopore sequencing data (-mode normal) [84]. We used GENC213 as a reference for ST113, GENC405 for ST53 and GENC414 for ST1503 (see Supplementary Data Set S1). The hybrid sequence of GENC200 was sequenced previously and was used as a reference for ST114 [19]. Mapping was carried out by using Snippy v3.2 [85]. The resulting core-SNPs were concatenated and used to infer ML trees for each ST analyzed by using IQ-tree and associated dependencies (–merit BIC: K3P+ASC –ufboot 1000 –bnni; intermediate) [68,69,70]. Finally, SNPs difference matrix files were generated after the removal of recombination elements with ClonalFrameML v1.12 [86].

### 4.6. Plasmid Profiles and Genetic Contents

Antibiotic resistance genes and plasmid replicons were detected with Abricate software v1.0.1 associated with ResFinder and PlasmidFinder databases, respectively (cut-off: ≥90.0% coverage and ≥70.0% nucleotide identity) [10,87,88]. For clinical isolates, ESBL-coding genes were predicted as associated with plasmids or chromosomes by combining different softwares (MOB-recon v3.0.3, PlasFlow v1.1.0, RFPlasmids v0.0.18) with default thresholds. The location was considered to be acceptable when at least two software programs produced the same result, with a prediction of >70.0%; other ESBL-carrying contigs were classified as “divergent” (see Supplementary Data Set S3) [11,12,13].

### 4.7. Sequencing and Synteny of a Major Local Resistance Plasmid

PlasmidFinder was used to identify an IncHI2 signature in 41 bacterial strains, of which 36 were clearly typed as ST1 (pMLST v2.0, IncHI2 DLST configuration, see Supplementary Table S3) [10]. This profile was described in the previously assembled and annotated pGENC200 plasmid (GenBank accession number CP061495) isolated from a local clinical ST114 strain [19,24]. We estimated the number of bases shared by the plasmid of new *Enterobacter* isolates and this first local reference by reconstructing putative plasmids by mapping trimmed reads with Bowtie 2 (v2.4.2) against pGENC200 [89]. Then, these selected mapped reads were assembled with SPAdes [74]. To assess the similarity of reconstructed elements, QUAST software (v5.0.2) was used against the local reference (Table S3) [75].

Next, to accurately determine the structural differences of the local IncHI2 mobile genetic elements, we focused on the fully reconstructed plasmids obtained by performing ONT long-read sequencing in Section 4.5 (pGENC213, pGENC405, pGENC414 and pGENC423; Table S3). The previously assembled and annotated pGENC200 plasmid was also analyzed [19]. Plasmid similarities were calculated by Mash distance according to the approach of Matlock et al. (*k-mer* length: 13; sketch size: 5000; Mash v2.1) [90,91]. Antibiotic resistance genes were detected with Abricate software as described above [87,88]. The PTU of this plasmid selection was identified using COPLA [21,22]. A syntenic analysis was performed against the larger circularized plasmid used as the reference (pGENC414: 349,057 bp). This step was performed with BRIG software v0.95 with default thresholds (Figure 3) [92]. The annotation of pGENC414 was performed on RAST server v2.0 (annotation scheme: RASTtk; Supplementary Data Set S3) [93]. The plasmid structures were compared and visualized with Mauve software v2.4.0 (Figure S4) [94].

### 4.8. Literature Search for Similar Plasmids

On 27 March 2022, in order to identify similar mobile genetic elements in the literature, the nucleotide sequence of pGENC414 was searched with Mash distance in the Plasmid database, PLSDB v2.1.1 (mash sketch -S 42 -k 21 -s 1000; max_*pvalue* = 0.1, max_dist = 0.1, individually = false) [23,91]. Only IncHI2/ST1 plasmids with complete metadata were conserved (associated bacterium, country, origin and submission date) [10]. In addition, the antibiotic resistance genes and PTU of these plasmids were identified as described above (see Supplementary Data Set S3) [21,87,88].

### 4.9. Global Phylogenetic Analysis of ECC Sutton’s Clade L

Because of the high prevalence of uncommon ESBL-producing strains belonging to the “UD4” *hsp60* cluster recovered in our study, initially associated with ECC Sutton’s clade L strains (Figure S3 and Supplementary Data Set S4) [3,18], and in order to place these isolates in the global context, all the WGS of ECC strains identified as belonging to this previous genogroup, with good quality and complete metadata (collection date, country and origin), were downloaded from GenBank on 29 June 2022 (see NCBI:txid2494701 and references [5,6]). Additional analyses were conducted with Similar Genome Finder to research similar public genomes in PATRIC resource center (v3.6.12 query reference: LEDN00000000, *p*-value: 0.001, Mash distance: ≤0.05) [3,91,95]. For quality control criteria, see Figure S6. Wild-type resistance pattern *hsp60* “UD4” GENC015 and GENC224 strains recovered from our clinical isolates (Figure S3) were also sequenced (Supplementary Data Set S4) [18].

The N50 mean of this ECC Sutton’s clade L collection (*n* = 58) was equal to 410,428 (min: 31,887, max: 5,111,427) [75]. The estimated mean BUSCO score was 98.8% completeness (single-copy; –lineage_Data Set_Enterobacterales; Supplementary Data Set S4) [76]. Antibiotic resistance genes and plasmid replicons were detected in this collection with Abricate associated with PlasmiFinder and ResFinder [10,87,88]. The ML phylogenetic tree was created as described above after annotation with Prokka and a global core-genome alignment with Roary [68,69,70,79,80]. The core-genome of the ECC Sutton’s clade L collection is composed of 3295 genes shared by at least 95.0% of the bacterial isolates, with a global alignment of 3,260,739 bp (Figure S5). We conducted another analysis after excluding all members of *E. chengduensis*, which was the least represented species from this clade, and conserved only ECC taxon 4 according to the approach of Wu et al. [5]. It corresponded to 38 isolates (Figure 4; 3539 genes shared by at least 95.0% of bacterial isolates, global alignment of 3,480,412 bp; –merit BIC: TIM2+F+R3 –ufboot 1000 –bnni) [68,69,70,79,80]. 

### 4.10. Data Analyses

Data collection and analyses were performed with Microsoft Access 2003. The incidence of ESBL-producer cases per 1000 patient days at the UHCG was calculated after sorting performed with GAM software for the management of medical, administrative and financial patient data. Pearson’s χ2 or Fisher’s exact tests were used to determine the significance of data (*p* < 0.05).

## 5. Conclusions

This study raises concern about emerging *Enterobacter* resistance species associated with a successful plasmid in Guadeloupe. Using various complementary sequencing approaches, we analyzed the genomic context of the *E. cloacae* complex between 2018 and 2019. The results for ESBL-producing strains suggest the dominance of specific lineages with intra-hospital dissemination and the wide diffusion of a well-conserved *bla*_CTX-M-15_/IncHI2/ST1 plasmid. By performing a comparison with international data, we demonstrated the local emergence of a novel sequence type. As several hospitalized patients have been infected with similar clones carrying large antibiotic resistance gene patterns, the strains should be compared with other strains and species collected from hospitals and community surveillance, especially in the Caribbean, to better appreciate regional differences and the possible spread of successful ESBL-producing lineages and associated plasmids; in addition, the screening of nosocomial environmental reservoirs should be performed.

## Data Availability

The authors confirm that all supporting data are provided with the article in the supplementary files. Antibiotic resistance profile results and partial *hsp60* coding sequences of the 135 clinical ECC strains are available on Supplementary Data Set S1. Eighty-nine partial *hsp60* coding sequences were obtained from previous supplementary material [18] and the others were newly sequenced. The 36 new whole-genome sequences of ECC isolates (ESBL producers, GENC015 and GENC224) and the four IncHI2/ST1 plasmids are deposited in GenBank database under BioProject no. PRJNA775655, while the 23 previously sequenced clinical isolates and plasmid (*n* = 1) were included in the PRJNA649757 and PRJNA659514 BioProjects. The corresponding accession numbers are listed in supplementary Data Set S1 for bacteria with ResFinder and PlasmidFinder outputs and Data Set S3 for plasmids. Additional information on *E. chengduensis* and *E. cloacae* complex taxon 4 analyses is presented in supplementary Data Set S4. Clinical metadata used for nosocomial incidence analyses are available in Supplementary Data Set S2.

## Supplementary Materials

The following supporting information can be downloaded at: https://www.mdpi.com/article/10.3390/antibiotics11101443/s1, Figure S1: Overall incidence of ESBL-producing *Enterobacteriaceae* at the University Hospital Center of Guadeloupe between 2018 and 2019 (*n* = 330); Figure S2: Distribution of *Enterobacter cloacae* complex strains according to the type of infection site; Figure S3: Maximum likelihood phylogenetic tree based on the partial hsp60 coding sequence of *E. cloacae* complex clinical isolates (*n* = 135); Figure S4: Alignment of the five analyzed *bla*_CTX-M-15_/IncHI2/ST1 plasmids; Figure S5: Maximum likelihood unrooted phylogenetic tree of *E. cloacae* complex Sutton’s clade L members; Figure S6: Schematic diagram of Materials and Methods—Section 4; Table S1: Prevalence of resistant *E. cloacae* complex (ECC) isolates for each tested antibiotic; Table S2: Abundance of identified resistance genes among the 57 *E. cloacae* complex (ECC) strains characterized as ESBL producers; and Table S3: Quast comparison of plasmids from previously sequenced pGENC200 (*E. xiangfangensis*, sequence type (ST) 114, CP061495) vs. reconstructed plasmids with an IncHI2 incompatibility group. The link includes also the following supplementary data set: Data Set S1: Sample metadata and details of genetic content of sequenced ESBL producer *E. cloacae* complex (*n* = 57); Data Set S2: Clinical incidence of hospital acquired ESBL-producing *Enterobacteriaceae* at the UHCG during the study period; Data Set S3: Metadata for the *bla*_CTX-M-15_/IncHI2/ST1 plasmids recovered in Guadeloupe; and Data Set S4: Metadata and details of genetic content of international collection of whole-genome sequenced and ESBL-producing *E. cloacae* complex Sutton’s clade L (*n* = 58); Data Set S5: Curated *Enterobacter* metadata in NCBI GenBank (1987–2019). References [3,4,5,10,11,12,13,18,19,24,26,63,67,68,69,70,71,72,74,75,77,78,80,87,88,89,94] are cited in the supplementary materials.
