# Supplementary material for: Emergence of a Novel Lineage and Wide Spread of a blaCTX-M-15/IncHI2/ST1 Plasmid among Nosocomial Enterobacter in Guadeloupe
Source: Antibiotics (Basel). 2022 Oct 20;11(10):1443. doi: 10.3390/antibiotics11101443 (PMC9598596; doi:10.3390/antibiotics11101443)
Supplement: Supplementary file 1 [file antibiotics-11-01443-s001.zip › antibiotics-1946755-supplementary.pdf]

antibiotics

MDPI

Emergence of a Novel Lineage and Wide Spread of a *bla*CTX-M-15/IncHI2/ST1 Plasmid among Nosocomial *Enterobacter* in Guadeloupe

Matthieu Pot [1,*], Yann Reynaud [1], David Couvin [1], Alexis Dereeper [1], Séverine Ferdinand [1], Sylvaine Bastian [2], Tania Foucan [3], Jean-David Pommier [4], Marc Valette [4], Antoine Talarmin [1], Stéphanie Guyomard-Rabenirina [1] and Sébastien Breurec [1,5,6]

1 Transmission, Reservoir and Diversity of Pathogens Unit, Pasteur Institute of Guadeloupe, 97139 Les Abymes, France
2 Laboratory of Clinical Microbiology, University Hospital Center of Guadeloupe, 97159 Pointe-à-Pitre, France
3 Operational Hygiene Team, University Hospital Center of Guadeloupe, 97159 Pointe-à-Pitre, France
4 Division of Intensive Care, University Hospital Center of Guadeloupe, 97159 Pointe-à-Pitre, France
5 Faculty of Medicine Hyacinthe Bastaraud, University of the Antilles, 97157 Pointe-à-Pitre, France
6 INSERM, Center for Clinical Investigation 1424, 97139 Les Abymes, France
* Correspondence: matthieu.pot@ird.fr

Supplementary Materials

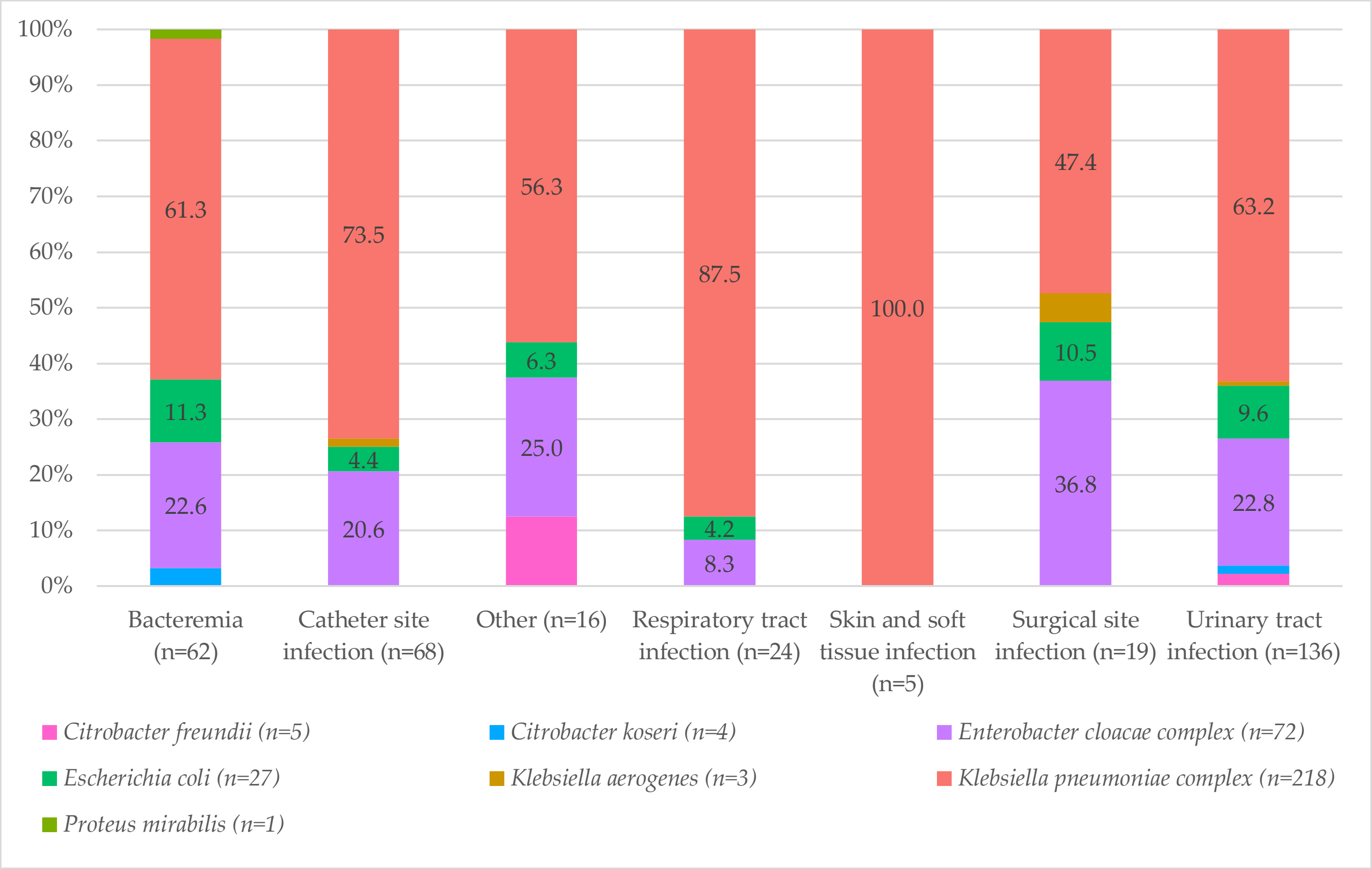

Figure S1. Overall incidence of ESBL-producing *Enterobacteriaceae* at the University Hospital Center of Guadeloupe between 2018 and 2019 (*n* = 330). These data were sorted using the GAM software for management of medical, administrative and financial patient data. The species were grouped according to the site of nosocomial infection. Only the prevalence of the three most represented species is shown on the graph (see details on Supplementary Data Set S2).

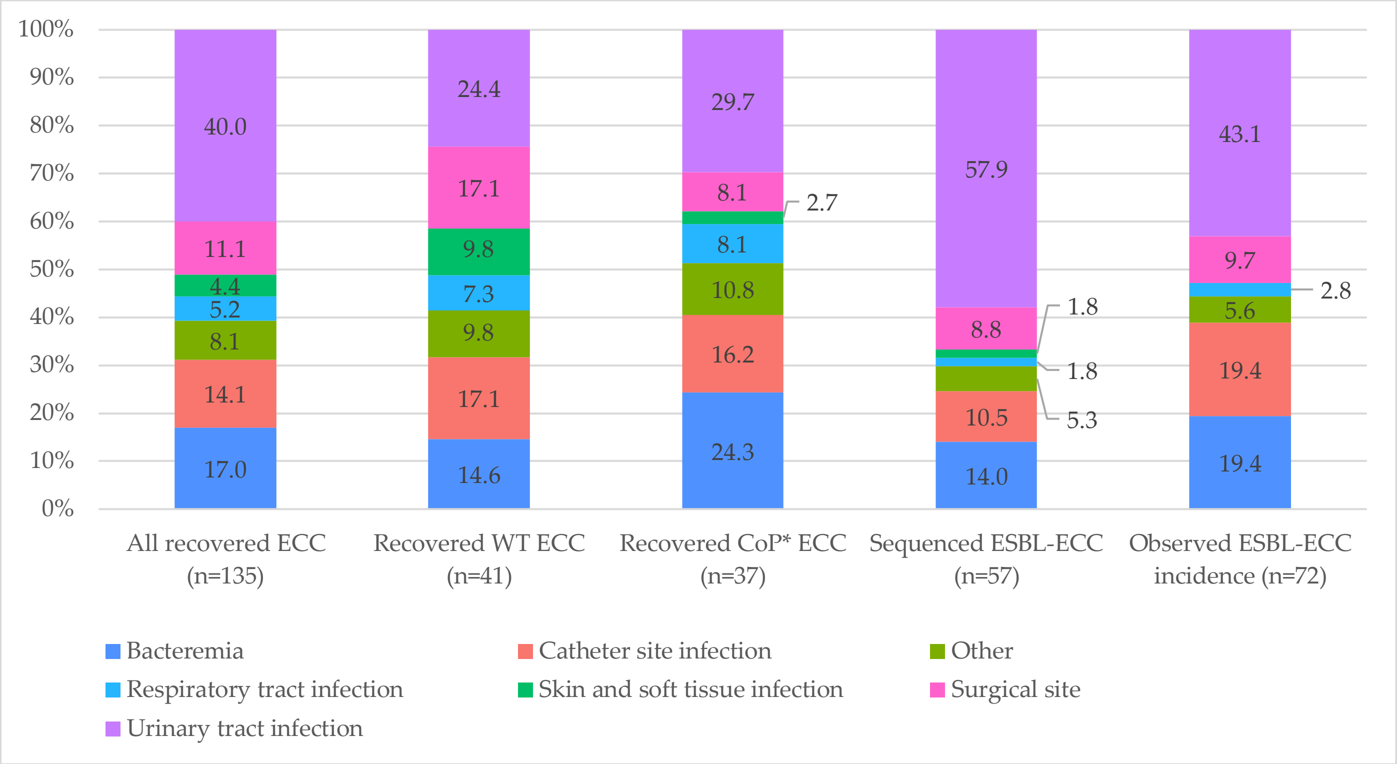

Figure S2. Distribution of *Enterobacter cloacae* complex strains according to the type of infection site. Recovered ECC isolates were grouped according to their resistance to β-lactam antibiotics: wild-type resistance profile (WT), sequenced ECC with extended-spectrum-β-lactamase (ESBL), and ECC with cephalosporinase overproduction without ESBL gene (CoP). * Among the CoP strains, one isolate was identified as a carbapenemase producer and was included on "Other" sample type. The observed ESBL-ECC incidence corresponds to data associated sorted using the GAM software for management of medical, administrative and financial patient data (see details on Supplementary Data Sets S1 and S2).

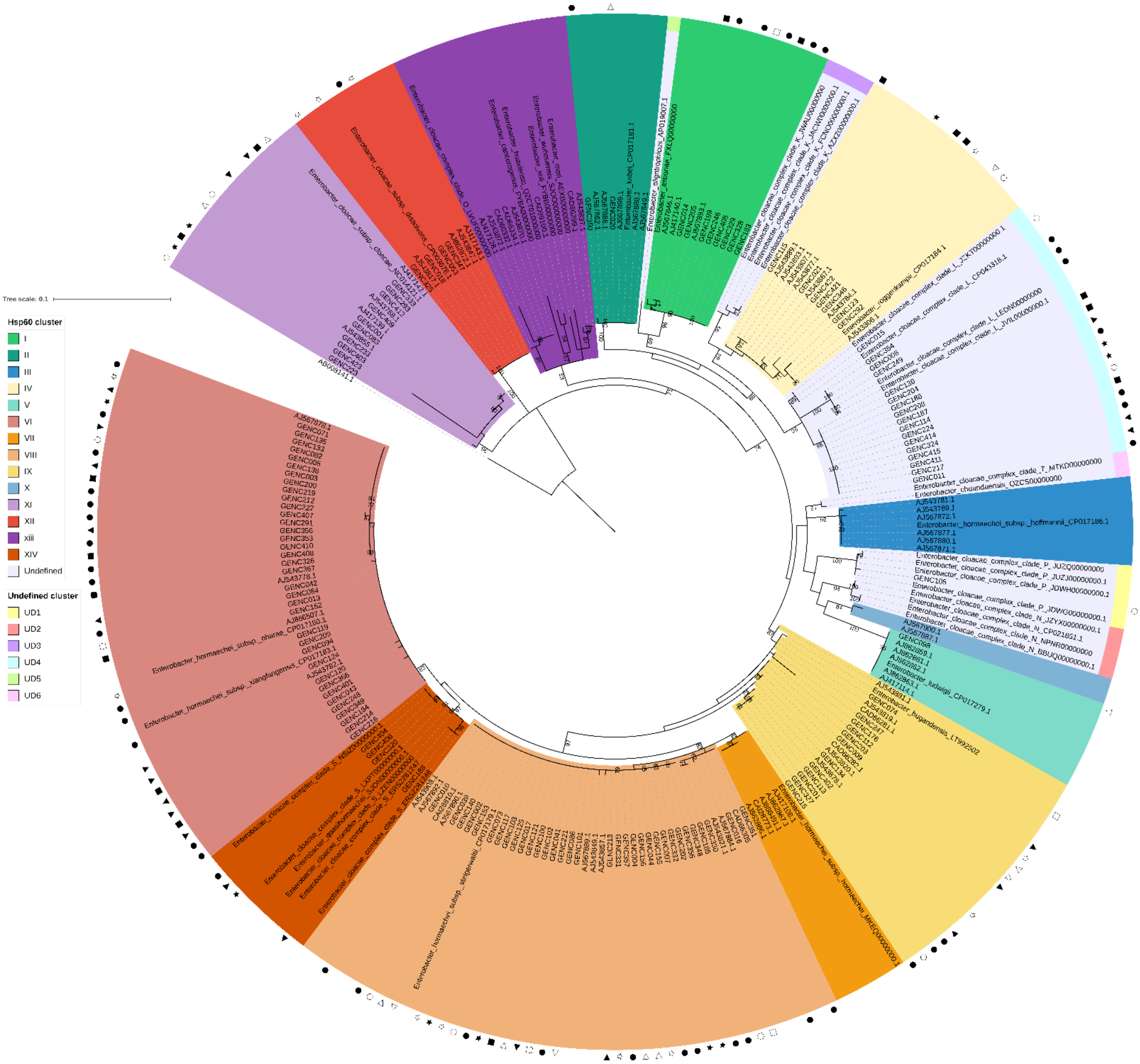

Figure S3. Maximum likelihood phylogenetic tree based on the partial hsp60 coding sequence of *E. cloacae* complex clinical isolates (*n* = 135). The tree drawn with iTOL v6.4.2 after maximum likelihood phylogenetic reconstruction with IQ-tree v2.1.2 and associated dependencies (–merit BIC: TIM3e+I+G4 –ufboot 1,000 –bnni), and sequence alignment with MAFFT (-flavor auto, v7.505) [1–5]. The 114 selected *E. cloacae* complex strains used as reference as described previously were labelled with the Sutton's nomenclature or species name, the *hsp60* cluster number, the whole-genome-related groups and their GenBank accession number [6–8]. A color was attributed to each cluster group, and only bootstrap values ≥ 60 are indicated. The numbers of the undefined (UD) *hsp60* cluster are the same as those used previously and cluster UD4 reference nodes were grouped for easier readability [8]. The most prevalent sample origins are sorted into four groups specified by the following symbols: circle (urine), square (blood), star (abscess, deep abscess, peritoneal liquid and pus), and left-pointing triangle (medical device: catheter); those with a right-pointing triangle are the least prevalent sample types, associated with respiratory tract infection (bronchial aspiration, bronchoalveolar lavage) and other infection sites. Antibiotic resistance profiles against β-lactam antibiotics are shown in white for wild-type strains and in black for third-generation cephalosporin-resistant strains. The global distribution, partial sequences and comparison with the hsp60EC-Ctool outputs are provided in Supplementary Data Set S1 [9].

List of ECC partial *hps60* sequence references included in this figure (associated cluster: C or UD, and accession number). This list is the same as the one used in our previous manuscript [8]:

C-I: AJ417140.1, AJ567846.1, AJ567893.1, FXLQ00000000; **C-II:** AJ567849.1, AJ567862.1, AJ567886.1, AJ567888.1, AJ567899.1, CP017181.1, **C-III:** AJ543781.1, AJ543789.1, AJ567871.1, AJ567872.1, AJ567877.1, AJ567880.1, CP017186.1; **C-IV:** AJ543784.1, AJ543806.1, AJ543807.1, AJ543867.1, AJ543877.1, AJ543889.1, AJ543893.1, CP017184.1; **C-V:** AJ417114.1, AJ862859.1, AJ862861.1, AJ862862.1, AJ862863.1, CP017279.1; **C-VI:** AJ543778.1, AJ543782.1, AJ567878.1, AJ866507.1, CP017180.1, CP017183.1; **C-VII:** AJ417108.1, AJ862866.1, AJ862867.1, AJ866491.1, CAI28773.1, MKEQ00000000.1; **C-VIII:** AJ543821.1, AJ543849.1, AJ543857.1, AJ543908.1, AJ567884.1, AJ567889.1, AJ567890.1, AJ567892.1, CAD66305, CAI28810.1, CP017179.1; **C-IX:** AJ543819.1, AJ543820.1, AJ543878.1, AJ543881.1, CAD66281.1, CAD66282.1, LT992502; **C-XI:** AJ417139.1, AJ417142.1, AJ543768.1, AJ543855.1, NC014121.1; **CXII:** AJ417143.1, AJ543817.1, AJ543847.1, AJ862872.1, CP003678.1; **C-XIII:** AJ417128.1, AJ543837.1, AJ543870.1, AJ543872.1, CAD66299.1, CAD66332.1, CAD66334.1, CAD99100.1, LVUF00000000, FYBB00000000, SJOO00000000, AEXB00000000, QZCT01000000, FYBA00000000; **C-XIV:** ERS2281247, ERS2281248, LXPT00000000.1, LZEN00000000, NSIZ00000000.1, JON00000000.1; **UD1:** JDWG00000000.1, JDWH00000000.1, JUZJ00000000.1, JUZQ00000000; **UD2:** BBUQ00000000.1, CP021851.1, JZYX00000000.1, NPNR00000000; **UD3:** AZXZ00000000.1, FCNO00000000.1, JACW00000000.1, JWAU00000000; **UD4:** CP043318.1, JVIL00000000.1, JZKT00000000.1, LEDN00000000; **UD5:** AP019007.1; **UD6:** MTKD00000000, QZCS00000000.

+ **C-X** (*Lelliottia nimipressuralis*, formerly *E. nimipressuralis*)

Supplementary Figure S3 is detailed on the following images (p 6 to 11):

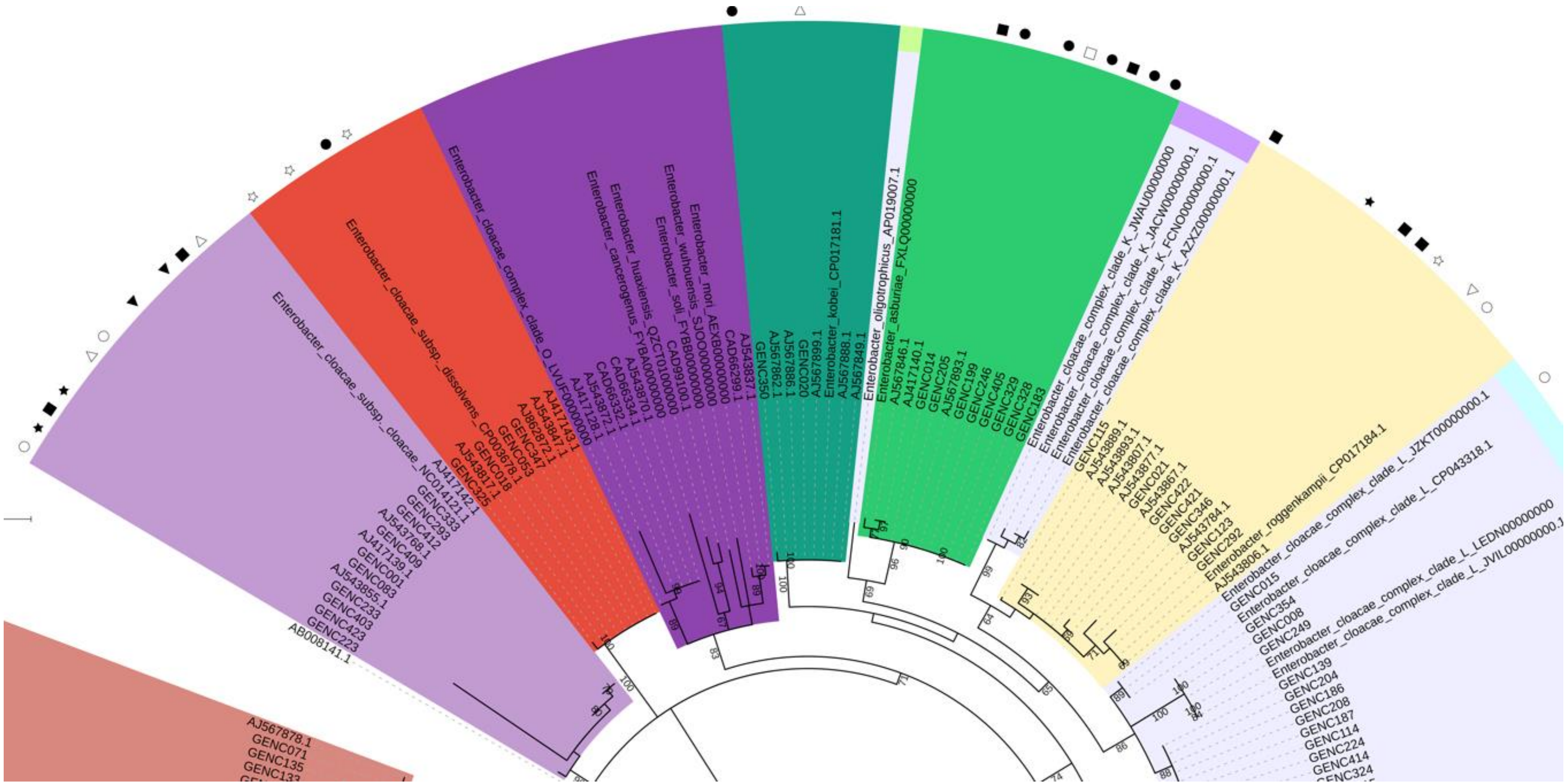

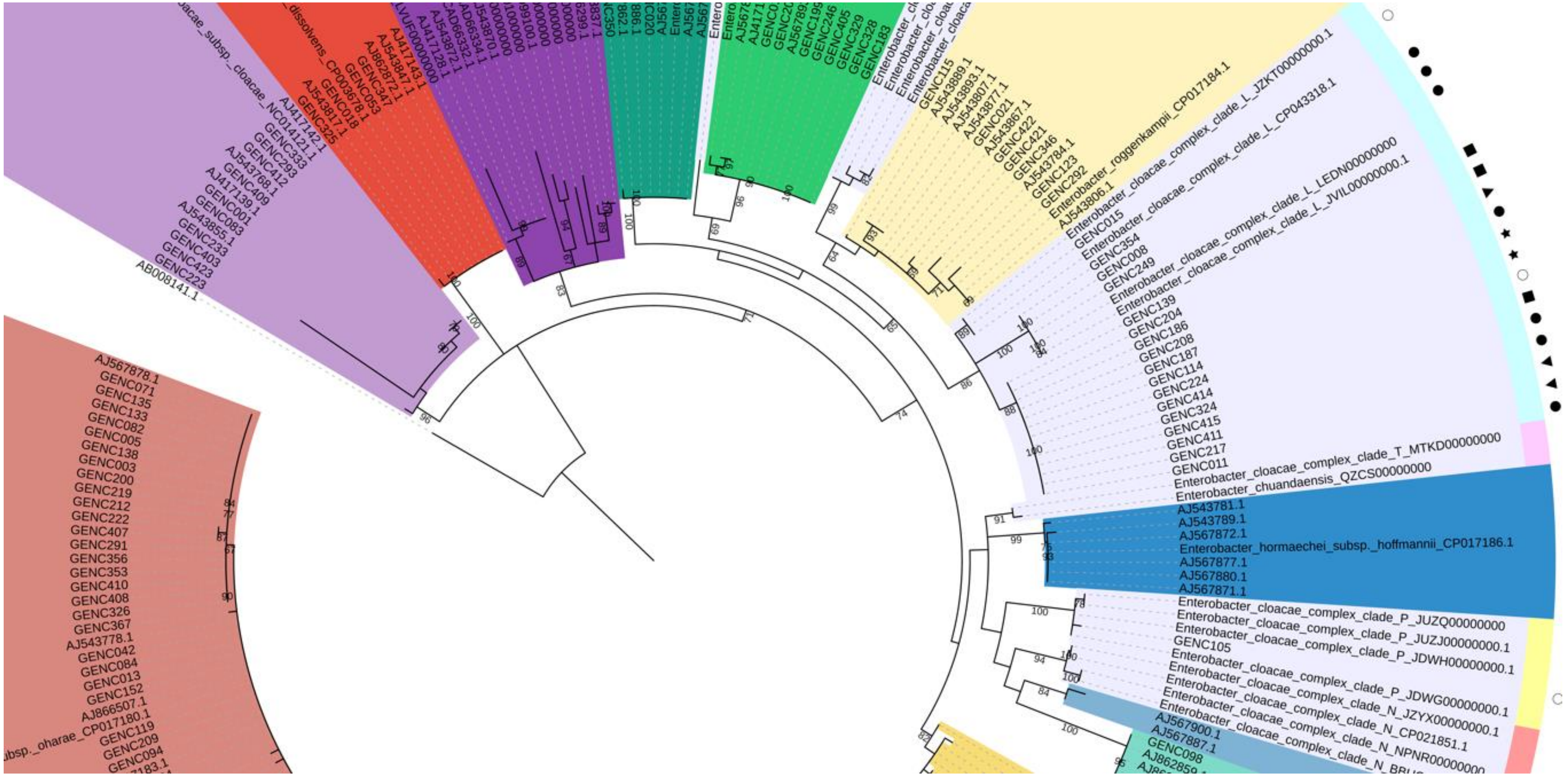
Enterobacter_roggenkampii_CP017184.1
Enterobacter_cloacae_complex_clade_L_JZKT00000000.1
Enterobacter_cloacae_complex_clade_L_CP043318.1
Enterobacter_cloacae_complex_clade_L_LEDN00000000
Enterobacter_cloacae_complex_clade_L_JVIL00000000.1
Enterobacter_cloacae_complex_clade_T_MTKD00000000
Enterobacter_chuandaensis_QZCS00000000
AJ543781.1
AJ543789.1
AJ567872.1
Enterobacter_hormaechei_subsp._hoffmannii_CP017186.1
AJ567877.1
AJ567880.1
AJ567871.1
Enterobacter_cloacae_complex_clade_P_JUZQ00000000
Enterobacter_cloacae_complex_clade_P_JUZJ00000000.1
GENC105
Enterobacter_cloacae_complex_clade_P_JDWH00000000.1
Enterobacter_cloacae_complex_clade_P_JDWG00000000.1
Enterobacter_cloacae_complex_clade_N_JZYX00000000.1
Enterobacter_cloacae_complex_clade_N_CP021851.1
Enterobacter_cloacae_complex_clade_N_NPNR00000000
AJ567878.1
GENC071
GENC135
GENC133
GENC082
GENC005
GENC138
GENC003
GENC200
GENC219
GENC212
GENC222
GENC407
GENC291
GENC356
GENC353
GENC410
GENC408
GENC326
GENC367
AJ543778.1
GENC042
GENC084
GENC013
GENC152
AJ866507.1
GENC119
GENC209
GENC094
AB008141.1
GENC139
GENC204
GENC186
GENC208
GENC187
GENC114
GENC224
GENC414
GENC324
GENC415
GENC411
GENC217
GENC011
AJ567900.1
AJ567887.1
GENC098

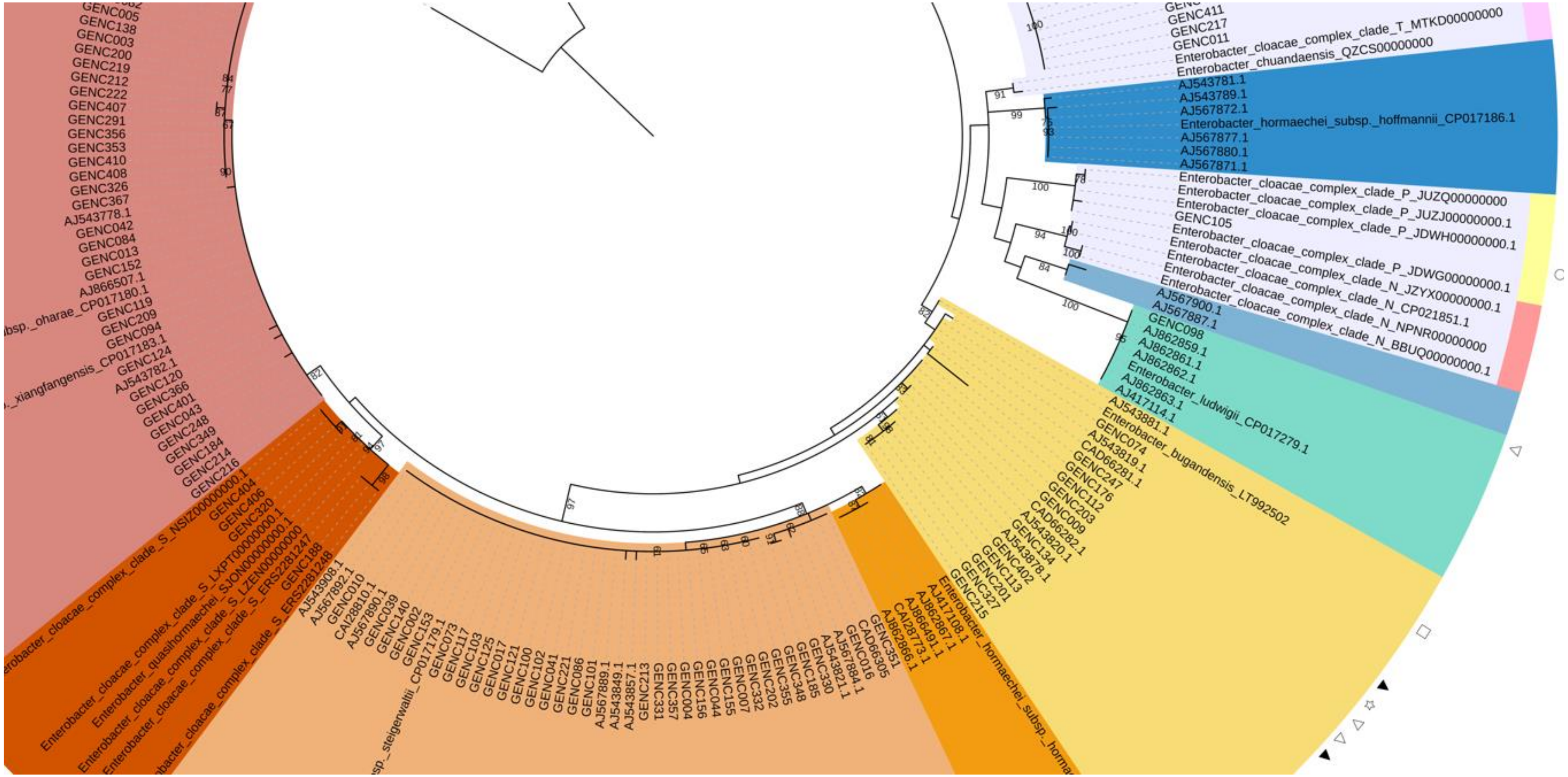
GENC411
GENC217
GENC011
Enterobacter_cloacae_complex_clade_T_MTKD00000000
Enterobacter_chuandaensis_QZCS00000000
AJ543781.1
AJ543789.1
AJ567872.1
Enterobacter_hormaechei_subsp._hoffmannii_CP017186.1
AJ567877.1
AJ567880.1
AJ567871.1
Enterobacter_cloacae_complex_clade_P_JUZQ00000000
Enterobacter_cloacae_complex_clade_P_JUZJ00000000.1
Enterobacter_cloacae_complex_clade_P_JDWH00000000.1
GENC105
Enterobacter_cloacae_complex_clade_P_JDWG00000000.1
Enterobacter_cloacae_complex_clade_N_JZYX00000000.1
Enterobacter_cloacae_complex_clade_N_CP021851.1
Enterobacter_cloacae_complex_clade_N_NPNR00000000
Enterobacter_cloacae_complex_clade_N_BBUQ00000000.1
AJ567900.1
AJ567887.1
GENC098
AJ862859.1
AJ862861.1
AJ862862.1
Enterobacter_ludwigii_CP017279.1
AJ862863.1
AJ417114.1
AJ543881.1
Enterobacter_bugandensis_LT992502
GENC074
AJ543819.1
CAD66281.1
GENC247
GENC176
GENC112
GENC203
GENC009
CAD66282.1
AJ543820.1
GENC134
AJ543878.1
GENC402
GENC113
GENC201
GENC327
GENC215
Enterobacter_hormaechei_subsp._hormae
AJ417108.1
AJ862867.1
AJ866491.1
CAI28773.1
AJ862866.1
GENC351
CAD66305
GENC016
AJ567884.1
AJ543821.1
GENC330
GENC185
GENC348
GENC355
GENC202
GENC332
GENC007
GENC155
GENC044
GENC156
GENC004
GENC357
GENC331
GENC213
AJ543857.1
AJ543849.1
AJ567889.1
GENC101
GENC086
GENC221
GENC041
GENC102
GENC100
GENC121
GENC017
GENC125
GENC103
GENC117
GENC073
sp._steigerwaltii_CP017179.1
GENC153
GENC002
GENC140
GENC039
AJ567890.1
CAI28810.1
GENC010
AJ567892.1
AJ543908.1
GENC188
Enterobacter_cloacae_complex_clade_S_ERS2281248
Enterobacter_cloacae_complex_clade_S_ERS2281247
Enterobacter_cloacae_complex_clade_S_LZEN00000000
Enterobacter_quasihormaechei_SJON00000000.1
Enterobacter_cloacae_complex_clade_S_LXPT00000000.1
GENC320
GENC406
GENC404
Enterobacter_cloacae_complex_clade_S_NSIZ00000000.1
GENC216
GENC214
GENC184
GENC349
GENC248
GENC043
GENC401
GENC366
GENC120
AJ543782.1
GENC124
_xiangfangensis_CP017183.1
GENC094
GENC209
GENC119
ubsp._oharae_CP017180.1
AJ866507.1
GENC152
GENC013
GENC084
GENC042
AJ543778.1
GENC367
GENC326
GENC408
GENC410
GENC353
GENC356
GENC291
GENC407
GENC222
GENC212
GENC219
GENC200
GENC003
GENC138
GENC005

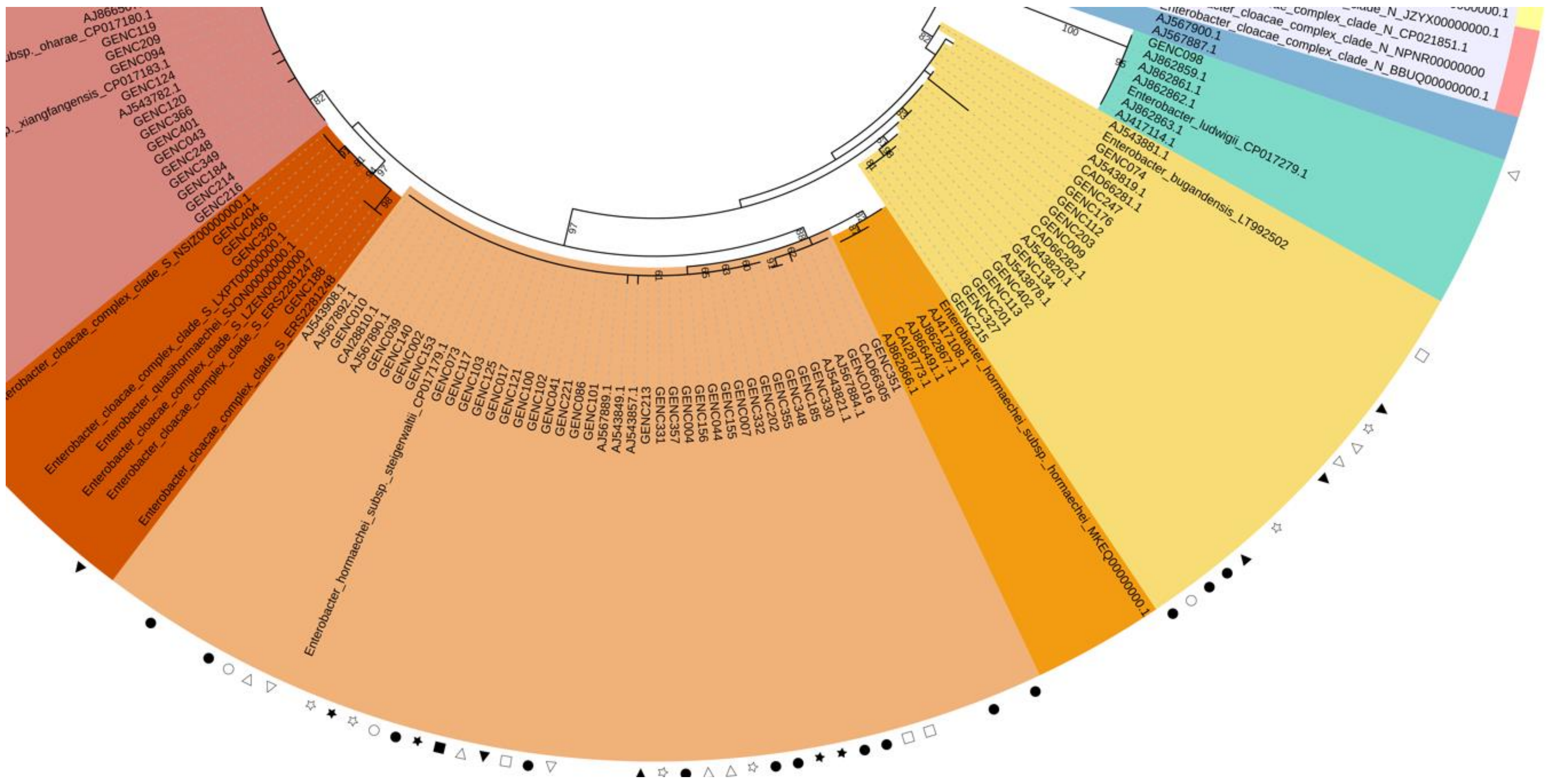

Enterobacter_cloacae_complex_clade_N_CP021851.1
Enterobacter_cloacae_complex_clade_N_NPNR00000000
Enterobacter_cloacae_complex_clade_N_BBUQ00000000.1
AJ567900.1
AJ567887.1
GENC098
AJ862859.1
AJ862861.1
AJ862862.1
Enterobacter_ludwigii_CP017279.1
AJ862863.1
AJ417114.1
AJ543881.1
Enterobacter_bugandensis_LT992502
GENC074
AJ543819.1
CAD66281.1
GENC247
GENC176
GENC112
GENC203
GENC009
CAD66282.1
AJ543820.1
GENC134
AJ543878.1
GENC402
GENC113
GENC201
GENC327
GENC215
Enterobacter_hormaechei_subsp._hormaechei_MKEQ00000000.1
AJ417108.1
AJ862867.1
AJ866491.1
CAI28773.1
AJ862866.1
GENC351
GENC305
CAD66305
GENC016
AJ567884.1
AJ543821.1
GENC330
GENC185
GENC348
GENC355
GENC202
GENC332
GENC007
GENC155
GENC044
GENC156
GENC004
GENC357
GENC331
GENC213
AJ543857.1
AJ543849.1
AJ567889.1
GENC101
GENC086
GENC221
GENC041
GENC102
GENC100
GENC121
GENC017
GENC125
GENC103
GENC117
GENC073
Enterobacter_hormaechei_subsp._steigerwaltii_CP017179.1
GENC153
GENC002
GENC140
GENC039
AJ567890.1
CAI28810.1
GENC010
AJ567892.1
AJ543908.1
GENC188
Enterobacter_cloacae_complex_clade_S_ERS2281248
Enterobacter_cloacae_complex_clade_S_ERS2281247
Enterobacter_cloacae_complex_clade_S_LZEN00000000
Enterobacter_quasihormaechei_SJON00000000.1
Enterobacter_cloacae_complex_clade_S_LXPT00000000.1
GENC320
GENC406
GENC404
Enterobacter_cloacae_complex_clade_S_NSIZ00000000.1
GENC216
GENC214
GENC184
GENC349
GENC248
GENC043
GENC401
GENC366
GENC120
AJ543782.1
GENC124
GENC094
GENC209
GENC119
AJ866507
100
97
98
82

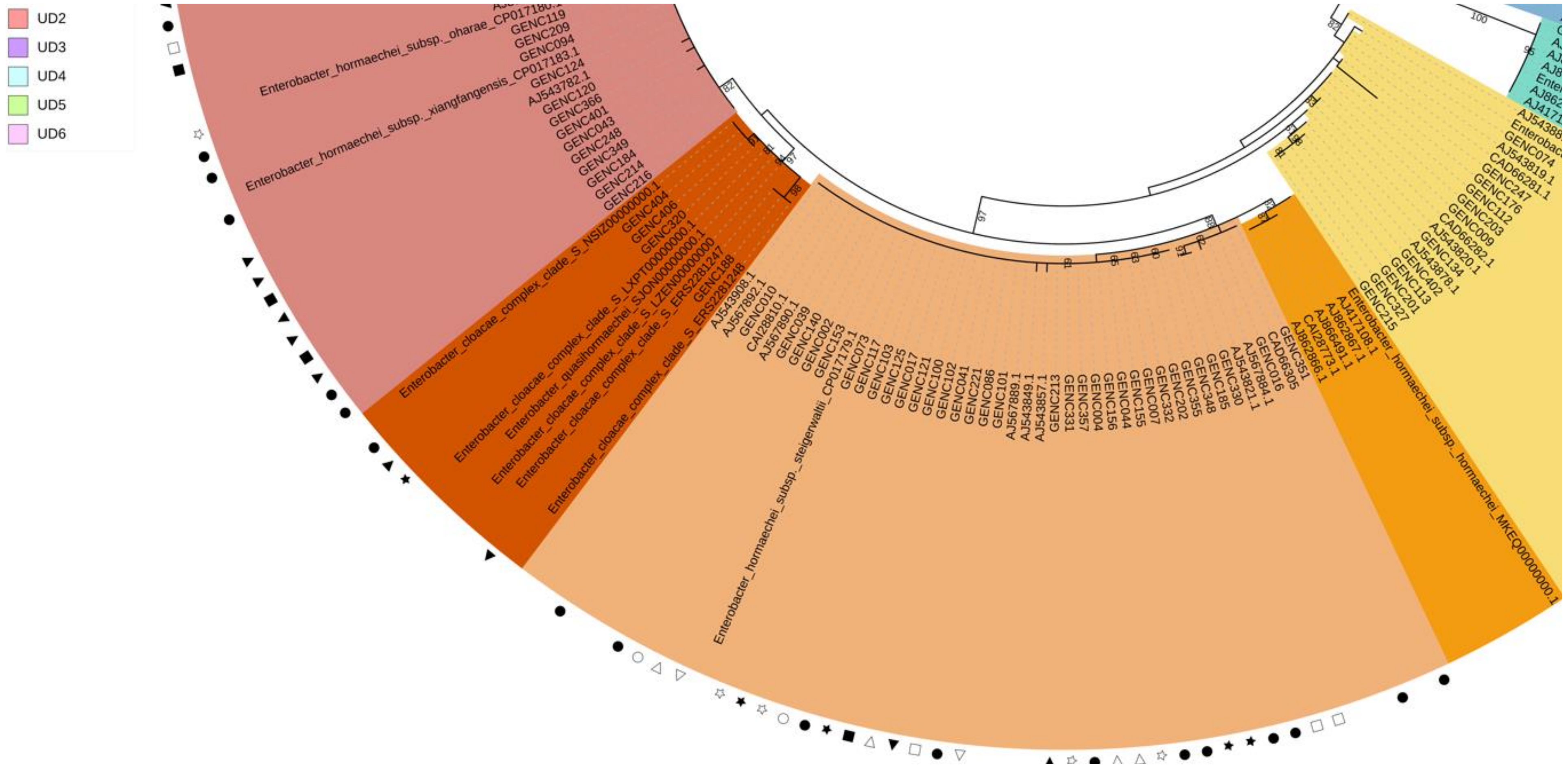
UD2
UD3
UD4
UD5
UD6
Enterobacter_hormaechei_subsp._oharae_CP017180.1
GENC119
GENC209
GENC094
Enterobacter_hormaechei_subsp._xiangfangensis_CP017183.1
GENC124
AJ543782.1
GENC120
GENC366
GENC401
GENC043
GENC248
GENC349
GENC184
GENC214
GENC216
Enterobacter_cloacae_complex_clade_S_NSIZ00000000.1
GENC404
GENC406
GENC320
Enterobacter_cloacae_complex_clade_S_LXPT00000000.1
Enterobacter_quasihormaechei_SJON00000000.1
Enterobacter_cloacae_complex_clade_S_LZEN00000000
Enterobacter_cloacae_complex_clade_S_ERS2281247
GENC188
Enterobacter_cloacae_complex_clade_S_ERS2281248
AJ543908.1
AJ567892.1
GENC010
CAI28810.1
AJ567890.1
GENC039
GENC140
GENC002
GENC153
Enterobacter_hormaechei_subsp._steigerwaltii_CP017179.1
GENC073
GENC117
GENC103
GENC125
GENC017
GENC121
GENC100
GENC102
GENC041
GENC221
GENC086
GENC101
AJ567889.1
AJ543849.1
AJ543857.1
GENC213
GENC331
GENC357
GENC004
GENC156
GENC044
GENC155
GENC007
GENC332
GENC202
GENC355
GENC348
GENC185
GENC330
AJ543821.1
AJ567884.1
GENC016
CAD66305
GENC351
AJ862866.1
CAI28773.1
AJ866491.1
AJ862867.1
AJ417108.1
Enterobacter_hormaechei_subsp._hormaechei_MKEQ00000000.1
GENC215
GENC327
GENC201
GENC113
GENC402
GENC134
AJ543878.1
AJ543820.1
CAD66282.1
GENC203
GENC009
GENC112
GENC176
GENC247
CAD66281.1
AJ543819.1
GENC074

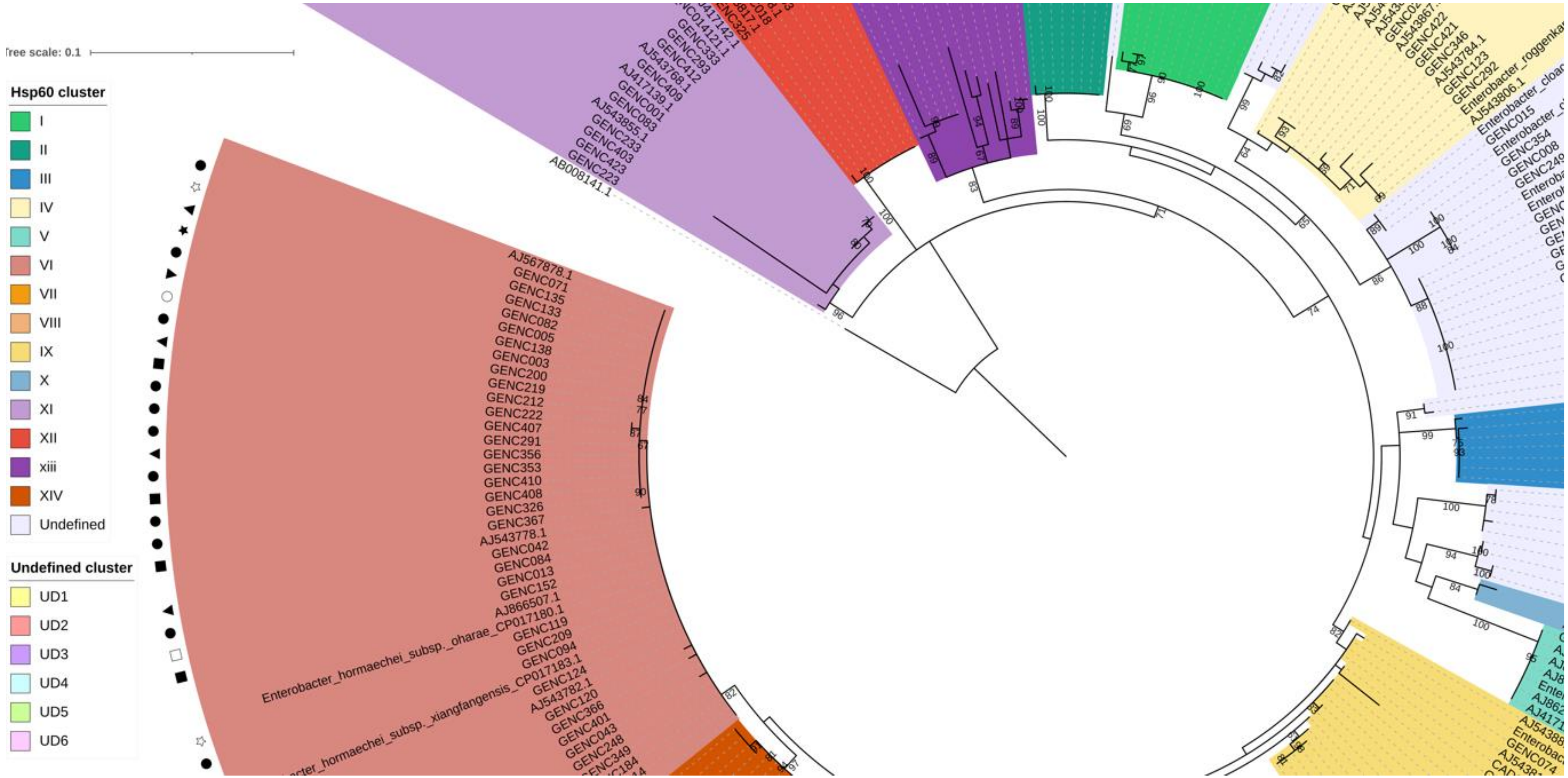
Tree scale: 0.1
Hsp60 cluster
I
II
III
IV
V
VI
VII
VIII
IX
X
XI
XII
xiii
XIV
Undefined
Undefined cluster
UD1
UD2
UD3
UD4
UD5
UD6
AJ567878.1
GENC071
GENC135
GENC133
GENC082
GENC005
GENC138
GENC003
GENC200
GENC219
GENC212
GENC222
GENC407
GENC291
GENC356
GENC353
GENC410
GENC408
GENC326
GENC367
AJ543778.1
GENC042
GENC084
GENC013
GENC152
AJ866507.1
Enterobacter_hormaechei_subsp._oharae_CP017180.1
GENC119
GENC209
GENC094
CP017183.1
GENC124
AJ543782.1
GENC120
GENC366
GENC401
GENC043
GENC248
GENC349
AB008141.1
GENC223
GENC423
GENC403
GENC233
AJ543855.1
GENC083
GENC001
AJ417139.1
GENC409
AJ543768.1
GENC412
GENC293
GENC333
GENC421
GENC346
AJ543784.1
GENC123
GENC292
AJ543806.1
GENC015
GENC354
GENC008
GENC074

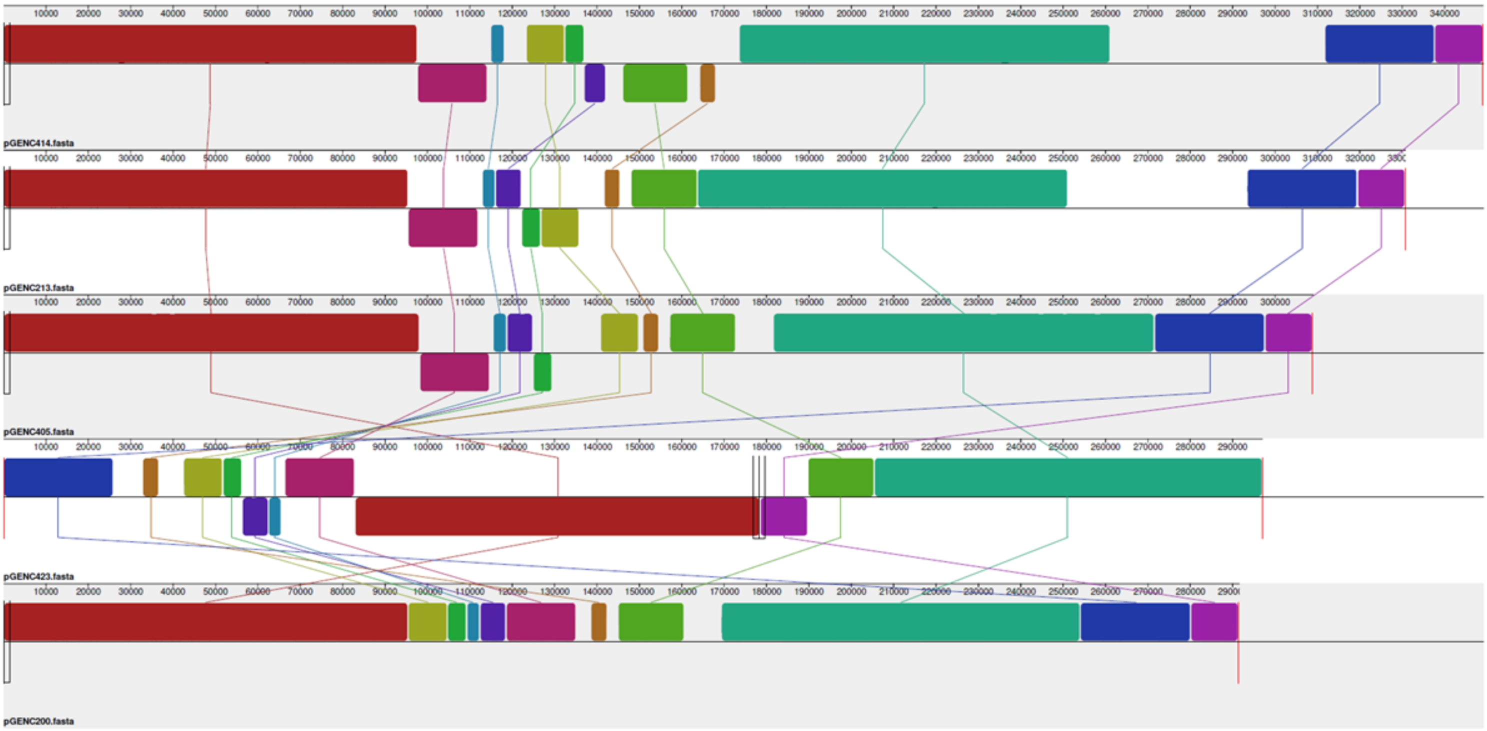

Figure S4. Alignment of the five analyzed *bla*$_{\text{CTX-M-15}}$/IncHI2/ST1 plasmids. Mauve software v2.4.0 was used to perform this alignment to visualize rearrangements and inversions within whole-sequenced plasmids from clinical ECC strains in Guadeloupe [10]. The plasmid order is based on their length: pGENC414 (349,057 bp; isolated from *E. cloacae* complex taxon 4), pGENC213 (330,805 bp; *E. xiangfangensis*), pGENC405 (308,992 bp; *E. asburiae*), pGENC423 (297,001 bp; *E. cloacae*) and pGENC200 (291,493 bp; *E xiangfangensis*).

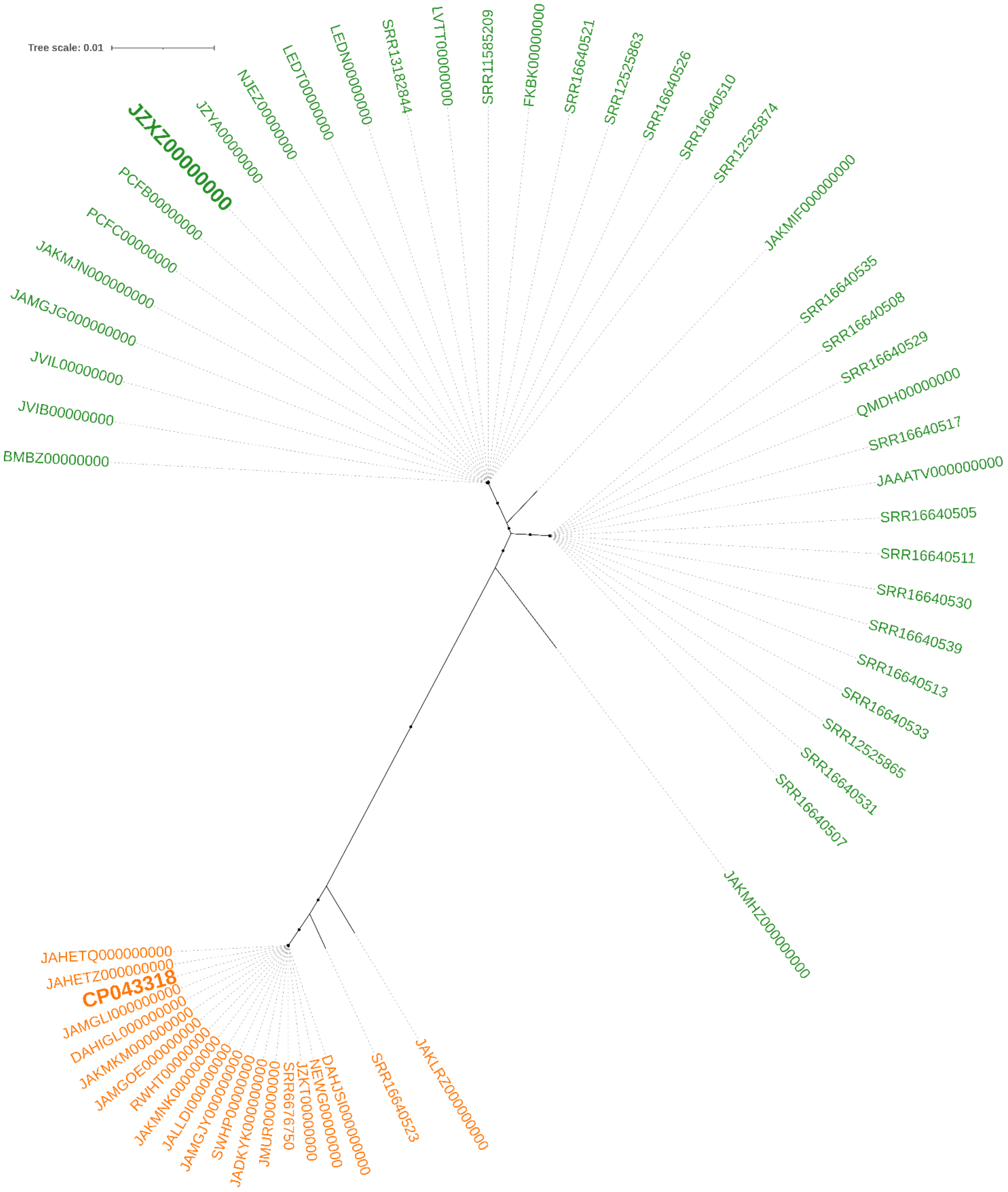

Figure S5. Maximum likelihood unrooted phylogenetic tree of *E. cloacae* complex Sutton's clade L members [7]. Sequences were downloaded from GenBank on 29 June 2022 [11]. This tree is based on the core-genome (-cd FLOAT 95: 3,295 genes; global alignment of 3,260,739 bp) and was drawn with iTOL after maximum likelihood phylogenetic reconstruction with IQ-tree and associated dependencies (–merit BIC: GTR+F+I+G4 –ufboot 1,000 –bnni) [1–4, 12]. Only bootstrap values equal to 100% are indicated by a black circle. According to Wu and colleagues approach, strains related to *E. cloacae* complex taxon 4 (reference in bold: JZXZ00000000) are in green (n = 38), while isolates of *E. chengduensis* (reference: CP043318) are in orange (n = 20) [13,14]. For associated ANI and isDDH outputs against references, see Supplementary Data Set S4 [7,13,15,16].

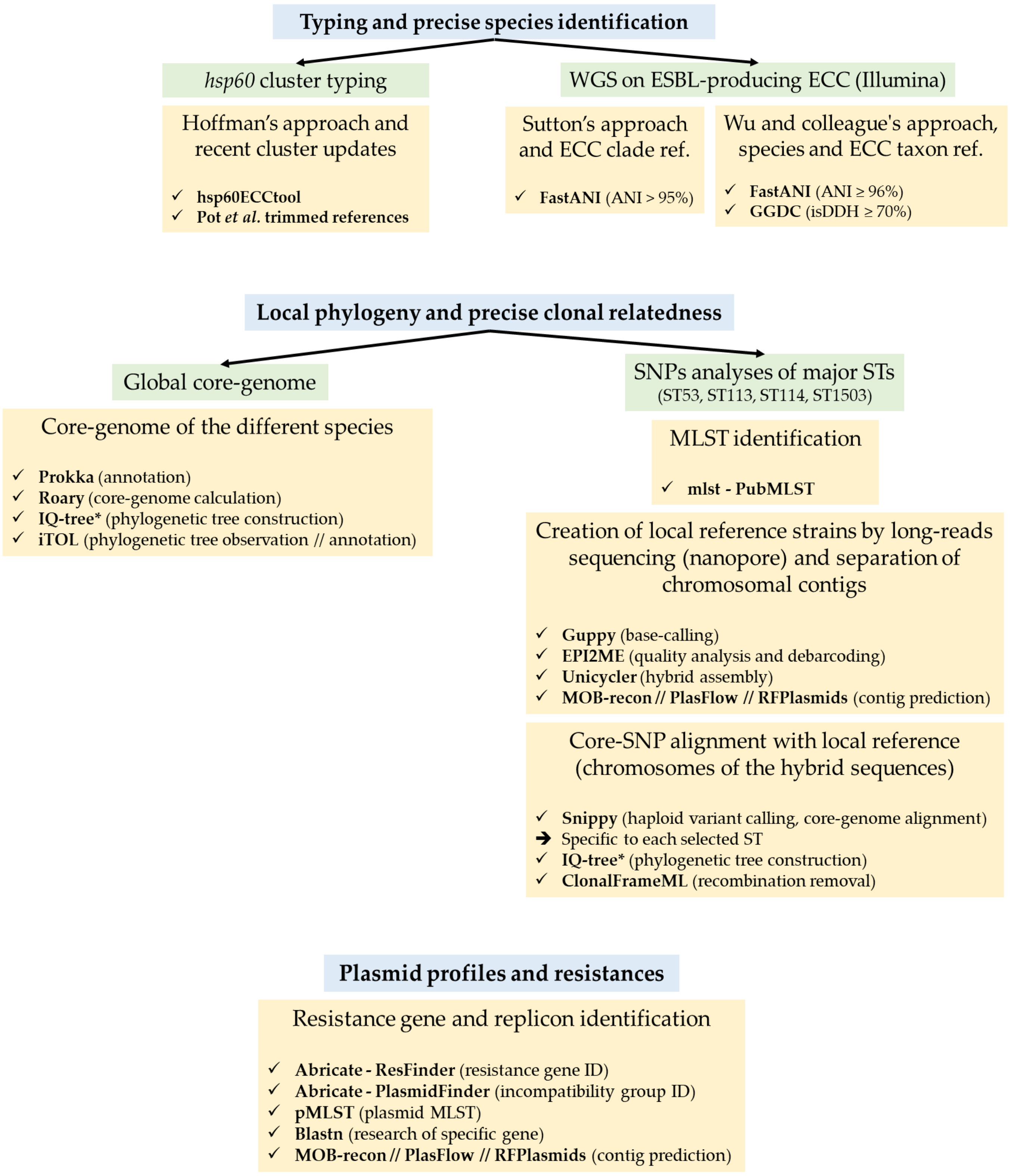

Figure S6. Continued on page 15

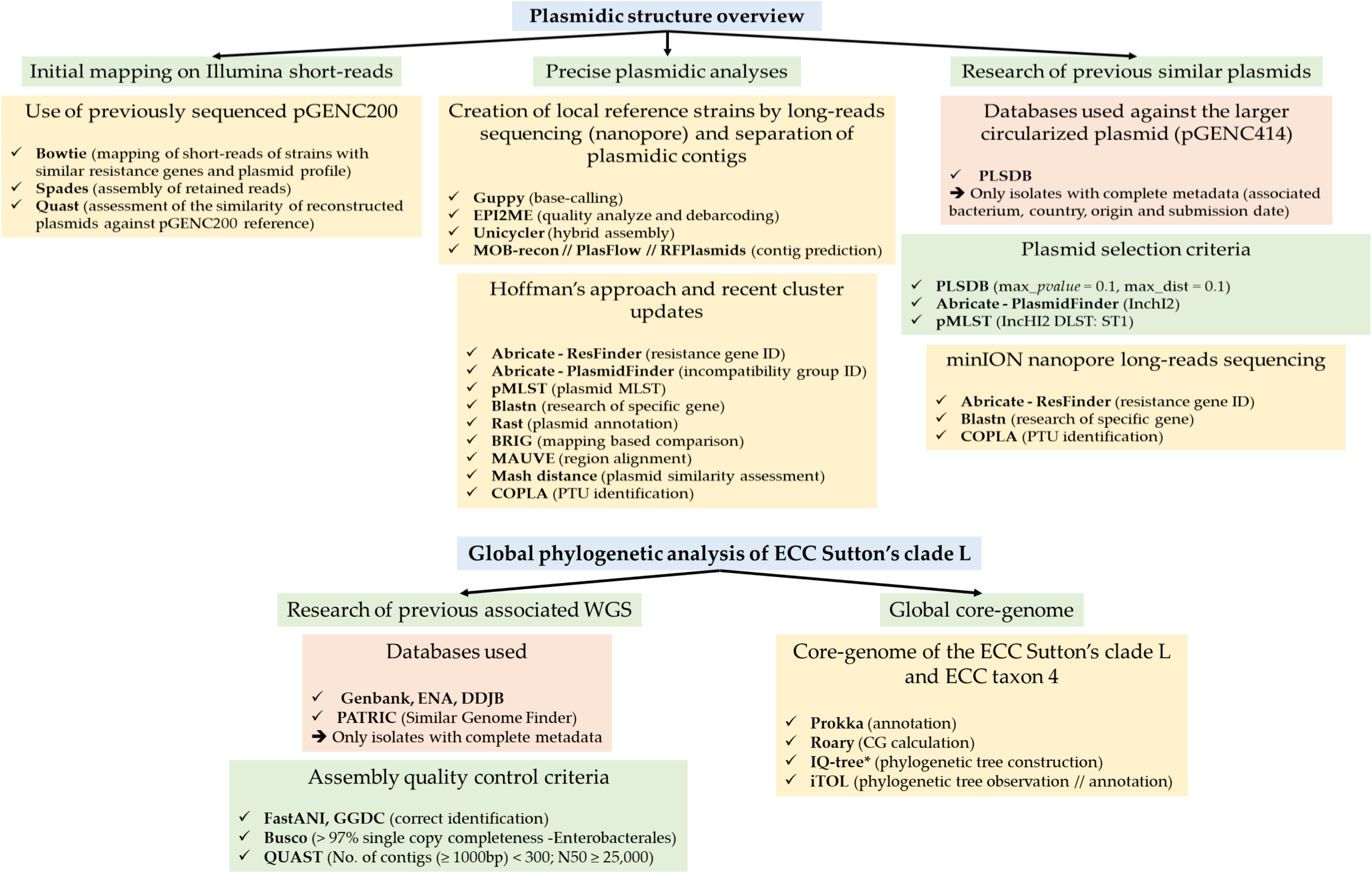

Figure S6. Schematic diagram of Materials and Methods – Section 4. Used software tools are indicated in bold and their applications in the study are in parentheses. *IQ-tree is associated with ModelFinder and UFBoot2. Pot *et al*. trimmed *hsp60* references were the same as those used in our previous manuscript [8]. They are listed on Figure S3 legend.

Table S1. Prevalence of resistant *E. cloacae* complex (ECC) isolates for each tested antibiotic. ECC strains (*n* = 135) were grouped according to their resistance to β-lactam antibiotics: wild-type (WT), extended spectrum β-lactamase (ESBL), cephalosporinase overproduction (CoP– without ESBL gene), or carbapenemase production (CP). All strains were resistant to ampicillin (10 µg), amoxicillin–clavulanic acid (20 µg – 10 µg) and cefoxitin (30 µg; see Supplementary Data Set S1 for details; disc diffusion method on Mueller-Hinton medium, 2018 CA-SFM/EUCAST guideline [17].

	**Resistance to β-lactam**			
**Antibiotic**	**WT** (***n*** **= 41)**	**CoP** (***n*** **= 36)**	**ESBL** (***n*** **= 57)**	**CP** (***n*** **= 1)**
Ticarcillin (75 µg)	1 (2.4)	36 (100.0)	57 (100.0)	1 (100.0)
Temocillin (30 µg)	–	30 (83.3)	31 (54.4)	1 (100.0)
Cefotaxime (5 µg)	–	36 (100.0)	57 (100.0)	1 (100.0)
Ceftazidime (10 µg)	–	34 (94.4)	57 (100.0)	1 (100.0)
Aztreonam (30 µg)	–	28 (77.8)	57 (100.0)	1 (100.0)
Ertapenem (10 µg)	–	17 (47.2)	3 (5.3)	1 (100.0)
Cefepim (30 µg)	–	–	57 (100.0)	1 (100.0)
Nalidixic acid (30 µg)	2 (4.9)	6 (16.7)	53 (93.0)	1 (100.0)
Ciprofloxacin (5 µg)	1 (2.4)	6 (16.7)	51 (89.5)	1 (100.0)
Gentamicin (10 µg)	–	3 (8.3)	45 (79.0)	–
Amikacin (30 µg)	–	–	1 (1.8)	–
Tigecycline (15 µg)	3 (7.3)	5 (13.9)	7 (12.3)	–
Trimethoprim – sulfamethoxazole (1.25 µg – 23.75 µg)	1 (2.4)	3 (8.3)	49 (86.0)	1 (100.0)

Table S2. Abundance of identified resistance genes among the 57 *E. cloacae* complex (ECC) strains characterized as ESBL producers. Strains were grouped at species level. The *mdf(A)* and *oqxA/oqxB* genes were identified in all strains as AmpC coding genes (not included in this table) [18,19]. Details are provided in Supplementary Data Set S1. **E.qah* = *E. quasihormaechei; *E. xian* = *E. xiangfangensis.*

Gene	*E. asburiae* **(*n* = 6)**	*E. bugandensis* **(*n* = 2)**	*E. cloacae* **(*n* = 2)**	*ECC Taxon 4* **(*n* = 15)**	*E. qah.** **(*n* = 1)**	*E. xian.*** **(*n* = 31)**	Total **(*n* = 57)**
*bla*CTX-M-15	6 (100.0)	2 (100.0)	2 (100.0)	15 (100.0)	1 (100.0)	30 (96.8)	56 (98.2)
dfrA14	5 (83.3)	–	1 (50.0)	15 (100.0)	1 (100.0)	30 (96.8)	52 (91.2)
fosA	1 (16.7)	2 (100.0)	2 (100.0)	15 (100.0)	1 (100.0)	30 (96.8)	51 (89.5)
qnrB1	5 (83.3)	–	1 (50.0)	14 (93.3)	1 (100.0)	28 (90.3)	49 (86.0)
aac(3)-IIa	5 (83.3)	–	–	13 (86.7)	1 (100.0)	26 (83.9)	45 (78.9)
aph(3'')-Ib	4 (66.7)	–	1 (50.0)	14 (93.3)	1 (100.0)	25 (80.6)	45 (78.9)
aph(6)-Id	4 (66.7)	–	1 (50.0)	14 (93.3)	1 (100.0)	25 (80.6)	45 (78.9)
sul2	4 (66.7)	–	1 (50.0)	14 (93.3)	–	25 (80.6)	44 (77.2)
*bla*TEM-1B	2 (33.3)	–	1 (50.0)	14 (93.3)	–	25 (80.6)	42 (73.7)
tet(A)	5 (83.3)	–	1 (50.0)	14 (93.3)	1 (100.0)	19 (61.3)	40 (70.2)
aac(6')-Ib-cr	4 (66.7)	–	2 (100.0)	11 (73.3)	–	19 (61.3)	36 (63.2)
catB3	4 (66.7)	–	1 (50.0)	11 (73.3)	–	20 (64.5)	36 (63.2)
*bla*OXA-1	4 (66.7)	–	1 (50.0)	11 (73.3)	–	19 (61.3)	35 (61.4)
sul1	3 (50.0)	–	1 (50.0)	4 (26.7)	1 (100.0)	14 (45.2)	23 (40.4)
catA1	3 (50.0)	–	–	3 (20.0)	–	13 (41.9)	19 (33.3)
dfrA1	–	–	–	–	–	5 (16.1)	5 (8.8)
aadA2	–	–	–	3 (20.0)	–	1 (3.2)	4 (7.0)
ant(3'')-Ia	–	–	–	2 (13.3)	–	2 (6.5)	4 (7.0)
qnrB19	1 (16.7)	–	–	1 (6.7)	–	2 (6.5)	4 (7.0)
mcr-9	–	–	–	–	–	2 (6.5)	2 (3.5)
aac(3)-I	–	–	–	–	–	1 (3.2)	1 (1.8)
aac(3)-Ia	–	–	–	–	–	1 (3.2)	1 (1.8)
aadA10	–	–	–	–	–	1 (3.2)	1 (1.8)
aadA16	–	–	1 (50.0)	–	–	–	1 (1.8)
aph(3')-VIa	–	–	–	1 (6.7)	–	–	1 (1.8)
ARR-3	–	–	1 (50.0)	–	–	–	1 (1.8)
*bla*GES-7	–	–	–	–	–	1 (3.2)	1 (1.8)
*bla*OXA-2	–	–	–	1 (6.7)	–	–	1 (1.8)
dfrA10	–	–	–	–	–	1 (3.2)	1 (1.8)
dfrA15	–	–	1 (50.0)	–	–	–	1 (1.8)
dfrA27	–	–	1 (50.0)	–	–	–	1 (1.8)
dfrA32	–	–	–	–	–	1 (3.2)	1 (1.8)
mph(E)	–	–	–	–	–	1 (3.2)	1 (1.8)
msr(E)	–	–	–	–	–	1 (3.2)	1 (1.8)
qnrE1	1 (16.7)	–	–	–	–	–	1 (1.8)
qnrS1	–	–	1 (50.0)	–	–	–	1 (1.8)
qnrS2	–	–	–	–	–	1 (3.2)	1 (1.8)
tet(C)	–	–	–	1 (6.7)	–	–	1 (1.8)
tet(D)	–	–	–	–	–	1 (3.2)	1 (1.8)

Table S3. Quast comparison of plasmids from previously sequenced pGENC200 (*E. xiangfangensis*, sequence type (ST) 114, CP061495) vs. reconstructed plasmids with an IncHI2 incompatibility group [20]. *E. cloacae* complex (ECC) plasmids were first reconstructed against the complete sequence of a locally representative plasmid (pGENC200) with Botwie2 (v2.4.2) and assembled with SPAdes (v3.12.0) [21–23]. Because of incomplete raw data, this analysis was not done for GENC010 and GENC016. Initial identification of incompatibility groups and plasmid typing was carried out with PlasmidFinder and pMLST (v2.0) databases [18,24]. Location prediction of the ESBL-coding gene by MOB-recon (v3.0.3), PlasFlow (v1.1.0) and RFPlasmids (v0.0.18) tools was divergent for two strains and predicted with only two types of software for only one isolate (*) [25–27]. Plasmid type was not defined for four strains (ND; For all details see Supplementary Data Set S1). Strains selected for long-read sequencing are specified with two stars.

Strain	WGS previous clade	Precise species identification	ST	Predicted *bla*$_{CTX-M-15}$ location	IncHI2 DLST conf.	Plasmidic reconstruction	
						Mapped length (bp)	% identity vs. pGENC200
**GENC353**	A	*E. xiangfangensis*	114	Plasmid	ST1	300,545	88.05
**GENC356**	A	*E. xiangfangensis*	114	Plasmid	ST1	348,301	90.68
**GENC326**	A	*E. xiangfangensis*	114	Plasmid	ST1	348,731	91.02
**GENC408**	A	*E. xiangfangensis*	114	Plasmid	ST1	419,303	91.03
**GENC212**	A	*E. xiangfangensis*	114	Plasmid	ST1	293,302	91.05
**GENC138**	A	*E. xiangfangensis*	114	Plasmid	ST1	266,170	91.14
**GENC071**	A	*E. xiangfangensis*	114	Plasmid	ST1	419,766	91.25
**GENC219**	A	*E. xiangfangensis*	114	Plasmid	ST1	398,055	91.27
**GENC291**	A	*E. xiangfangensis*	114	Chromosome	ST1	295,215	93.07
**GENC410**	A	*E. xiangfangensis*	114	Plasmid	ST1	295,623	94.02
**GENC005**	A	*E. xiangfangensis*	114	Plasmid	ST1	281,763	96.18
**GENC082**	A	*E. xiangfangensis*	114	Chromosome	ST1	291,977	96.43
**GENC214**	A	*E. xiangfangensis*	171	Chromosome	ND	293,064	82.71
**GENC184**	A	*E. xiangfangensis*	171	Chromosome	ND	292,593	83.06
**GENC152**	A	*E. xiangfangensis*	544	Plasmid	ST1	294,402	94.42
**GENC094**	A	*E. xiangfangensis*	544	Plasmid	ST1	331,971	95.05
**GENC209**	A	*E. xiangfangensis*	–	Plasmid	ST1	319,223	92.31
**GENC202**	B	*E. xiangfangensis*	45	Plasmid	ST1	296,246	93.53
**GENC039**	B	*E. xiangfangensis*	113	Plasmid	ST1	262,592	90.04
**GENC017**	B	*E. xiangfangensis*	113	Plasmid	ST1	274,488	94.03
**GENC155**	B	*E. xiangfangensis*	113	Plasmid	ST1	293,740	94.33
**GENC213** **	B	*E. xiangfangensis*	113	Plasmid	ST1	293,871	95.03
**GENC351**	B	*E. xiangfangensis*	133	Chromosome	ST1	294,508	96.47
**GENC007**	B	*E. xiangfangensis*	190	Plasmid	ST1	278,479	95.35
**GENC423** **	G	*E. cloacae*	167	Plasmid*	ST1	304,198	93.87
**GENC199**	J	*E. asburiae*	53	Divergent	ND	295,301	95.74
**GENC183**	J	*E. asburiae*	53	Divergent	ST1	294,168	96.20
**GENC405** **	J	*E. asburiae*	53	Plasmid	ST1	294,851	96.51
**GENC354**	L	ECC taxon 4	598,1799	Plasmid	ST1	301,774	88.12
**GENC249**	L	ECC taxon 4	598,1799	Plasmid	ND	294,606	95.36
**GENC008**	L	ECC taxon 4	598,1799	Plasmid	ST1	294,984	96.25
**GENC324**	L	ECC taxon 4	1503	Plasmid	ST1	298,878	93.18
**GENC415**	L	ECC taxon 4	1503	Plasmid	ST1	294,336	93.75
**GENC217**	L	ECC taxon 4	1503	Plasmid	ST1	298,999	94.05
**GENC411**	L	ECC taxon 4	1503	Plasmid	ST1	294,385	94.18
**GENC139**	L	ECC taxon 4	1503	Plasmid	ST1	298,027	94.20
**GENC187**	L	ECC taxon 4	1503	Plasmid	ST1	294,553	94.68
**GENC414** **	L	ECC taxon 4	1503	Plasmid	ST1	298,854	94.76

References

1. Letunic, I.; Bork, P. Interactive Tree of Life (iTOL) v5: An online tool for phylogenetic tree display and annotation. *Nucleic Acids Res.* **2021**, *49*, W293–W296. https://doi.org/10.1093/nar/gkab301.
2. Nguyen, L.T.; Schmidt, H.A.; von Haeseler, A.; Minh, B.Q. IQ-TREE: A fast and effective stochastic algorithm for estimating maximum-likelihood phylogenies. *Mol. Biol. Evol.* **2015**, *32*, 268–274. https://doi.org/10.1093/molbev/msu300.
3. Hoang, D.T.; Chernomor, O.; von Haeseler, A.; Minh, B.Q.; Vinh, L.S. UFBoot2: Improving the ultrafast bootstrap approximation. *Mol. Biol. Evol.* **2018**, *35*, 518–522. https://doi.org/10.1093/molbev/msx281.
4. Kalyaanamoorthy, S.; Minh, B.Q.; Wong, T.K.F.; von Haeseler, A.; Jermiin, L.S. ModelFinder: Fast model selection for accurate phylogenetic estimates. *Nat. Methods.* **2017**, *14*, 587–589. https://doi.org/10.1038/nmeth.4285.
5. Katoh, K.; Standley, D.M. MAFFT multiple sequence alignment software version 7: Improvements in performance and usability. *Mol. Biol. Evol.* **2013**, *30*, 772–780. https://doi.org/10.1093/molbev/mst010.
6. Hoffmann, H.; Roggenkamp, A. Population genetics of the nomenspecies *Enterobacter cloacae*. *Appl. Environ. Microbiol.* **2003**, *69*, 5306–5318. https://doi.org/10.1128/AEM.69.9.5306-5318.2003.
7. Sutton, G.G.; Brinkac, L.M.; Clarke, T.H.; Fouts, D.E. *Enterobacter hormaechei* subsp. *hoffmannii* subsp. nov., *Enterobacter hormaechei* subsp. *xiangfangensis* comb. nov., *Enterobacter roggenkampii* sp. nov., and *Enterobacter muelleri* is a later heterotypic synonym of *Enterobacter asburiae* based on computational analysis of sequenced *Enterobacter* genomes. *F1000Research* **2018**, *7*, 521. https://doi.org/10.12688/f1000research.
8. Pot, M.; Reynaud, Y.; Couvin, D.; Ducat, C.; Ferdinand, S.; Gravey, F.; Gruel, G.; Guérin, F.; Malpote, E.; Breurec, S.; et al. Wide distribution and specific Resistance pattern to third-generation cephalosporins of *Enterobacter cloacae* complex members in humans and in the environment in Guadeloupe (French West Indies). *Front. Microbiol.* **2021**, *12*, 628058. https://doi.org/10.3389/fmicb.2021.628058.
9. GitHub – hsp60ECCtool. Available online: https://github.com/karubiotools/hsp60ECCtool (accessed on 9 November 2021).
10. Darling, A.C.; Mau, B.; Blattner, F.R.; Perna, N.T. Mauve: Multiple alignment of conserved genomic sequence with rearrangements. *Genome Res.* **2004**, *14*, 1394–1403. https://doi.org/10.1101/gr.2289704.
11. NCBI Resource Coordinators. Database resources of the National Center for Biotechnology Information. *Nucleic Acids Res.* **2016**, *44*, D7–D19. https://doi.org/10.1093/nar/gkv1290.
12. Page, A.J.; Cummins, C.A.; Hunt, M.; Wong, V.K.; Reuter, S.; Holden, M.T.; Fookes, M.; Falush, D.; Keane, J.A.; Parkhill, J. Roary: Rapid large-scale prokaryote pan genome analysis. *Bioinformatics* **2015**, *31*, 3691–3693. https://doi.org/10.1093/bioinformatics/btv421.
13. Wu, W.; Feng, Y.; Zong, Z. Precise species identification for *Enterobacter*: A genome sequence-based study with reporting of two novel species, *Enterobacter quasiroggenkampii* sp. nov. and *Enterobacter quasimori* sp. nov. *mSystems* **2020**, *5*, e00527-20. https://doi.org/10.1128/mSystems.00527-20.
14. Wu, W.; Feng, Y.; Zong, Z. Characterization of a strain representing a new *Enterobacter* species, *Enterobacter chengduensis* sp. nov. *Antonie Van Leeuwenhoek* **2019**, *112*, 491–500. https://doi.org/10.1007/s10482-018-1180-z.
15. Jain, C.; Rodriguez, R.L.M.; Phillippy, A.M.; Konstantinidis, K.T.; Aluru, S. High throughput ANI analysis of 90K prokaryotic genomes reveals clear species boundaries. *Nat. Commun.* **2018**, *9*, 5114. https://doi.org/10.1038/s41467-018-07641-9.
16. Meier-Kolthoff, J.P.; Carbasse, J.S.; Peinado-Olarte, R.L.; Göker, M. TYGS and LPSN: A database tandem for fast and reliable genome-based classification and nomenclature of prokaryotes. *Nucleic Acids Res.* **2022**, *50*, D801–D807. https://doi.org/10.1093/nar/gkab902.
17. Société Française de Microbiologie. *CA-SFM / EUCAST: Comité de L'antibiogramme de la Société Française de Microbiologie—Recommandation 2018*; Société Française de Microbiologie: Paris, France, 2018; pp. 1–132.
18. GitHub – Abricate. Available online: https://github.com/tseemann/abricate (accessed on 19 April 2020).
19. Florensa, A.F.; Kaas, R.S.; Clausen, P.T.L.C.; Aytan-Aktug, D.; Aarestrup, F.M. ResFinder—An open online resource for identification of antimicrobial resistance genes in next-generation sequencing data and prediction of phenotypes from genotypes. *Microb. Genom.* **2022**, *8*, 000748. https://doi.org/10.1099/mgen.0.000748.
20. Gurevich, A.; Saveliev, V.; Vyahhi, N.; Tesler, G. QUAST: Quality assessment tool for genome assemblies. *Bioinformatics* **2013**, *29*, 1072–1075. https://doi.org/10.1093/bioinformatics/btt086.
21. Pot, M.; Guyomard-Rabenirina, S.; Couvin, D.; Ducat, C.; Enouf, V.; Ferdinand, S.; Gruel, G.; Malpote, E.; Talarmin, A.; Breurec, S.; et al. Dissemination of extended-spectrum-β-Lactamase-producing *Enterobacter cloacae* complex from a hospital to the nearby environment in Guadeloupe (French West Indies): ST114 Lineage coding for a successful IncHI2/ST1 plasmid. *Antimicrob. Agents Chemother.* **2021**, *65*, e02146-20. https://doi.org/10.1128/AAC.02146-20.
22. Prjibelski, A.; Antipov, D.; Meleshko, D.; Lapidus, A.; Korobeynikov, A. Using SPAdes *de novo* assembler. *Curr. Protoc. Bioinform.* **2020**, *70*, e102. https://doi.org/10.1002/cpbi.102.
23. Langmead, B.; Salzberg, S.L. Fast gapped-read alignment with Bowtie 2. *Nat Methods.* **2012**, *9*, 357–359. https://doi.org/10.1038/nmeth.1923.
24. Carattoli, A.; Zankari, E.; García-Fernández, A.; Voldby Larsen, M.; Lund, O.; Villa, L.; Møller Aarestrup, F.; Hasman, H. In silico detection and typing of plasmids using PlasmidFinder and plasmid multilocus sequence typing. *Antimicrob. Agents Chemother.* **2014**, *58*, 3895–3903. https://doi.org/10.1128/AAC.02412-14.
25. Krawczyk, P.S.; Lipinski, L.; Dziembowski, A. PlasFlow: Predicting plasmid sequences in metagenomic data using genome signatures. *Nucleic Acids Res.* **2018**, *46*, e35. https://doi.org/10.1093/nar/gkx1321.

26. van der Graaf-van Bloois, L.; Wagenaar, J.A.; Zomer, A.L. RFPlasmid: Predicting plasmid sequences from short-read assembly data using machine learning. *Microb. Genom.* **2021**, *7*, 000683. https://doi.org/10.1099/mgen.0.000683.
27. Robertson, J.; Nash, J.H.E. MOB-suite: Software tools for clustering, reconstruction and typing of plasmids from draft assemblies. *Microb. Genom.* **2018**, *4*, e000206. https://doi.org/10.1099/mgen.0.000206.
